# Influential upregulation of KCNE4: Propelling cancer associated fibroblasts-driven colorectal cancer progression

**DOI:** 10.1186/s12935-024-03274-9

**Published:** 2024-03-10

**Authors:** Zizhen Zhang, Shengde Liu, Zhenghang Wang, Shuo Wang, Lei Jiang, Xicheng Wang, Jian Li, Lin Shen

**Affiliations:** 1 Key Laboratory of Carcinogenesis and Translational Research (Ministry of Education/Beijing), Department of Gastrointestinal Oncology, Peking University Cancer Hospital & Institute, Beijing, 100142 China; 2 Key Laboratory of Colorectal Cancer Diagnosis and Treatment Research, Department of Gastroenterological Surgery, Peking University People’s Hospital, Beijing, 100044 China

**Keywords:** Colorectal cancer (CRC), Cancer-associated fibroblasts (CAFs), KCNE4, Adhesion, Migration

## Abstract

**Background:**

Colorectal cancer (CRC) is a malignancy of remarkable heterogeneity and heightened morbidity. Cancer associated fibroblasts (CAFs) are abundant in CRC tissues and are essential for CRC growth. Here, we aimed to develop a CAF-related classifier for predicting the prognosis of CRC and identify critical pro-tumorigenic genes in CAFs.

**Method:**

The mRNA expression and clinical information of CRC samples were sourced from two comprehensive databases, The Cancer Genome Atlas (TCGA) and Gene Expression Omnibus (GEO). Using a weighted gene co-expression network analysis (WGCNA) approach, CAF-related genes were identified and a CAF risk signature was developed through the application of univariate analysis and the least absolute shrinkage and selection operator (LASSO) Cox regression model. EdU cell proliferation assay, and transwell assay were performed to detect the oncogenic role of KCNE4 in CAFs.

**Results:**

We constructed a prognostic CAF model consisting of two genes (SFRP2 and KCNE4). CRC patients were classified into low- and high-CAF-risk groups using the median CAF risk score, and patients in the high-CAF-risk group had worse prognosis. Meanwhile, a higher risk score for CAFs was associated with greater stromal and CAF infiltrations, as well as higher expression of CAF markers. Furthermore, TIDE analysis indicated that patients with a high CAF risk score are less responsive to immunotherapy. Our further experiments had confirmed the strong correlation between KCNE4 and the malignant phenotypes of CAFs. Moreover, we had shown that KCNE4 could actively promote tumor-promoting phenotypes in CAFs, indicating its critical role in cancer progression.

**Conclusion:**

The two-gene prognostic CAF signature was constructed and could be reliable for predicting prognosis for CRC patients. Moreover, KCNE4 may be a promising strategy for the development of novel anti-cancer therapeutics specifically directed against CAFs.

**Supplementary Information:**

The online version contains supplementary material available at 10.1186/s12935-024-03274-9.

## Introduction

Colorectal cancer (CRC) is a prevalent and fatal malignancy of the gastrointestinal tract worldwide [1, 2]. The development of metastasis to distant organs is strongly associated with a reduced 5-year survival rate and lower quality of life [3, 4]. Therefore, it is crucial to develop effective strategies to prevent CRC metastasis. CRC is a complex disease that involves not only neoplastic cancer cells but also a diverse array of immune and stromal cells that coexist within the tumor microenvironment (TME) [5, 6]. The TME is a dynamic and heterogeneous milieu that plays a crucial role in the initiation, progression, and metastasis of CRC, ultimately determining the tumor’s biological behavior, which has a direct bearing on the patient’s prognosis [7, 8]. Therefore, understanding the complexity and heterogeneity of the TME in CRC is essential for the development of effective therapies that could target both the cancer cells and their microenvironment.

Cancer-associated fibroblasts (CAFs), which constitute the primary constituents of the tumor stroma, serve as significant regulators that modulate tumor migration and progression, promote epithelial–mesenchymal transition (EMT), and cause both chemoresistance and immunosuppression [7, 9]. Functionally, CAFs secrete extracellular matrix components that shape the TME, creating a physical barrier against immune cell infiltration, which results in a reduction of cytotoxic immune cell infiltration, enabling tumor immune escape [9, 10]. Additionally, CAFs secrete immunosuppressive cytokines facilitating the formation of an immunosuppressive microenvironment, further impairing immune cell function and promoting immune escape [11, 12].

Currently, CAFs are generally classified into myofibroblast CAFs (myoCAFs) and inflammatory CAFs (iCAFs) [13–15]. MyoCAFs are mainly located around tumor cells and are involved in the formation of the extracellular matrix [16]. On the other hand, iCAFs secrete various cytokines and chemokines that can act on tumor cells [15]. However, some targeted therapy studies aimed at CAFs have surprisingly revealed that removing CAFs may actually promote tumor progression or metastasis, implying a significant degree of heterogeneity within the CAFs population [17, 18]. Hence, targeting the CAFs-mediated immunosuppressive stromal microenvironment, in conjunction with immunotherapy, could be a viable strategy for improving the response to immune checkpoint inhibitors (ICI). Whereas, numerous clinical trials targeting CAFs, including those that targeted FAP protein expressed in CAFs cells, have yielded unsatisfactory results, highlighting the pressing need for identifying a specific CAFs marker that can be leveraged as a therapeutic target [19, 20].

Through the utilization of RNA-seq data obtained from TCGA and GEO datasets, our study adopted the systematic bioinformatics algorithm WGCNA to identify the hub modules most strongly correlated with stromal CAFs infiltration. Subsequently, we employed univariate and LASSO Cox regression analyses to screen for potential prognostic CAFs markers, and discovered KCNE4 and SFRP2 as robust candidates. We then developed a two-gene signature based on these markers, which effectively predicts clinical outcome and therapeutic response in patients with CRC. Potassium Voltage-Gated Channel Subfamily E Regulatory Subunit 4 (KCNE4) was identified and subsequently validated as a potential biomarker candidate through our rigorous investigation.

Of the two pivotal genes, KCNE4’s role in CAFs remains largely unexplored. Here, we report for the first time that overexpression of KCNE4 in normal fibroblasts drives the conversion of NAFs to CAFs and enhances tumor cell metastasis. Consequently, targeting KCNE4 can reverse the malignant properties of CAFs and offer a potential therapeutic avenue for CRC.

## Materials and methods

### Data acquisition

The TCGA RNA-seq data in fragments per kilobase of transcript per million mapped reads (FPKM) format, along with the corresponding clinical data of TCGA colon and rectal cancer (TCGA-CRC) samples, were acquired through the UCSC Xena browser (GDC hub) at https://gdc.xenahubs.net. The gene profiles and clinical data of 177 CRC samples in GSE17536 [21] were acquired by downloading the dataset from the GEO project.

### The calculation of stromal score and estimation of CAF infiltration

To quantify the degree of stromal infiltration within each individual tumor specimen, we employed the Estimation of Stromal and Immune cells in Malignant Tumor tissues (ESTIMATE) algorithm and the advanced estimate R package (version 1.0.13) to calculate the stromal score [22]. Moreover, four distinct methodologies were employed to accurately quantify the abundance of CAFs, thus improving our understanding of their crucial function within the TME. The Estimate the Proportion of Immune and Cancer cells (EPIC) algorithm, leveraging constrained least square optimization, enabled precise cell-type deconvolution [23]. Additionally, the xCell algorithm harnessed gene signature enrichment analysis and distinguished different subsets of CAFs populations [24]. The microenvironment cell populations-counter (MCP-counter) algorithm based on the expression of specific marker genes was used to obtain more detailed information on CAFs landscape [25]. These methods were implemented using the deconvolute () function from the immunedeconv R package (version 2.0.3) [26]. Moreover, the Tumor Immune Dysfunction and Exclusion (TIDE) method was seamlessly implemented through the user-friendly interface accessible at http://tide.dfci.harvard.edu/ [27].

EPIC: Focuses on estimating cell fractions in tumor samples using reference gene expression profiles from blood and tumor-infiltrating cells, covering a comprehensive range of nonmalignant cell types found in human tumors, including immune, stromal, and endothelial cells. It aims for precise absolute cell fraction predictions [28].

xCell: A gene signature-based approach that deduces the presence of 64 different immune and stromal cell types by integrating 1822 human cell type transcriptomes from various sources. xCell employs a curve-fitting comparison method and introduces a spillover compensation technique to accurately differentiate between cell types [24].

MCP-Counter: Offers robust quantification of eight immune and two stromal cell populations in heterogeneous tissues via transcriptomic data, producing abundance scores for specific cell types. It facilitates direct comparisons of cell type abundance across samples within a study, functioning on a per-sample basis [25].

TIDE: Simulates tumor immune evasion mechanisms by analyzing the roles of T cell dysfunction and barriers to T cell infiltration, based on gene expression and its impact on patient survival. TIDE predicts immunotherapy outcomes more accurately than traditional biomarkers and identifies potential regulators of immune checkpoint blockade resistance [27].

### The induction of CAF and stromal co-expression network

The WGCNA R package (version 1.72) was utilized to construct co-expression networks and identify hub genes that targeted CAFs infiltrations and stromal scores [29]. For both the TCGA-CRC and GSE17536 cohorts, the top 5,000 genes were selected from the MAD for input gene selection. Utilizing the topological overlap measure (TOM) and dissimilarity (1-TOM) between genes, genes were identified in the adjacency matrix. Subsequently, a dynamic tree cut algorithm was applied to the dendrogram to detect modules of co-expressed genes. A subset of hub genes was identified for further analysis by overlapping the most correlated module genes between the TCGA-CRC and GSE17536 cohorts. This subset showed the highest correlation between module eigengenes and EPIC-quantified cancer-associated fibroblast infiltrations, as well as the stromal score.

## The analysis of the kyoto encyclopedia of genes and genomes and gene ontology

The biological functions of the hub genes were analyzed using the clusterProfiler R package (version 3.14.3). This analysis included evaluation of molecular functions (MFs), biological processes (BPs), cellular components (CCs), and pathways based on GO and KEGG databases [30]. Statistics were considered significant when *p* < 0.05.

### Predictive algorithm development and verification

The TCGA cohort was used to construct the CAF risk model, while the GEO cohort was utilized as the validation cohort. Prognostic CAF hub genes for overall survival (OS) were identified through performing univariate Cox regression analysis. To decrease the number of genes, a LASSO Cox regression analysis was conducted with 1,000 iterations using the glmnet R package for genes with *p* < 0.05 [31]. An individual’s CAF risk score is calculated as ∑ (β_i_ * Exp_i_), where β_i_ is the LASSO coefficient of ith gene, and Exp_i_ is its expression. The CRC patients’ cohort was divided into high- and low-CAF risk groups based on their respective median CAF scores to establish the CAF risk model. The distinction in OS between these groups was evaluated using Kaplan-Meier curves and a log-rank test.

### Somatic alteration data collection, analysis, and enrichment analyses

The somatic mutation data of the TCGA-CRC cohort were acquired using the GDCquery Maf () function with the “mutect2” [32] pipeline of the TCGAbiolinks R package [33]. In both the low-CAF-risk and high-CAF-risk groups, the top 20 genes with the highest mutational frequencies were determined. The comparison of KEGG pathway gene sets between high- and low-CAF-risk groups in TCGA-CRC dataset was performed using Gene Set Enrichment Analysis (GSEA). The “c2.cp.kegg.v7.4.symbols” gene sets from MSigDB were employed [34]. Additionally, ssGSEA [35] was employed to estimate the enrichment scores for the gene sets related to Focal adhesion, TGF-β, ECM receptor interaction, and Regulation of actin cytoskeleton. The association between CAF risk score and gene set enrichment scores was assessed using Spearman’s correlation analysis.

### The outcomes of chemotherapy and immunotherapy prediction

Genomics of Drug Sensitivity in Cancer [GDSC] database (https://www.cancerrxgene.org/) [36] was used to estimate half-maximal inhibitory concentration (IC50) values of common drugs in each CRC sample based on the transcriptome data by ridge regression through ten-fold cross-validation in pRRophetic R package (version 0.5) [37]. The TIDE online algorithm at http://tide.dfci.harvard.edu/ was utilized to predict the response to immune checkpoint blockade therapy [27]. The predictive performance of the CAF risk signature was evaluated using ROC curves and the corresponding area under the curves (AUC).

### Cancer cell line Encyclopedia (CCLE) validation

To confirm our cellular-level observations, mRNA expression profiles of the identified markers were procured from the CCLE database (https://portals.broadinstitute.org/ccle) [38] for both fibroblasts and CRC cell lines. Heatmaps and Wilcoxon tests were utilized to scrutinize the expression patterns of these markers in both cell types.

### Clinical samples

Fresh tissue samples were obtained from patients with CRC at Peking University Cancer Hospital. Tissue samples were frozen in -80℃ for following the research. The Ethics Committee of Peking University Cancer Hospital approved all studies involving clinical samples.

### Isolation and culture of CAFs

Fresh human CRC tissue and adjacent normal tissue were procured from Peking University Cancer Hospital for this investigation. Prior to uniformly compressing the CRC tissue onto a culture plate, fragments of 2 mm diameter were excised. CAFs were permitted to migrate using 20% FBS-DMEM medium at 37 °C and 5% CO_2_ for a period of 1–2 weeks, following which they were purified via enzymatic digestion. Subsequently, western blotting was carried out to confirm the expression of FAP, FSP1 and α-SMA in the CAFs.

### Cell lines

The LoVo, HCT116 and RKO cell lines were obtained from the Chinese Academy of Medical Sciences (Beijing, China), and HEK293T cells were obtained from the American Type Culture Collection (ATCC). Cells were cultured in DMEM medium (Gibco) with 10% fetal bovine serum (FBS) (Gibco) and 1% penicillin-streptomycin at 37 °C with 5% CO_2_.

### Western blot

Fresh tumor and adjacent non-cancerous tissues from CRC patients were homogenized using a Servicebio homogenizer (KZ-III-FP) in Thermo Fisher Scientific RIPA lysis buffer (89,900). Tissue was homogenized, incubated on ice for 30 min, centrifuged at 12,000 rpm, and mixed with 5× loading buffer. Boiling the sample for 10 min at 100 °C prepared it for subsequent analysis. Samples were transferred to PVDF membranes (0.22 μm) after SDS-PAGE and overnight incubated with primary antibodies. HRP-conjugated secondary antibodies were then added, and the membranes visualized using enhanced chemiluminescence. Experiments were independently conducted three times. Antibodies utilized in the experiment were as follows: anti-KCNE4 (1:1,000; ER61021), anti-α-SMA (1:1000; ET1612-13), anti-FAP (1:1000; ET1704-23), anti-Vimentin (1:1000; ET1610-39), anti-FSP1 (1:200; ET1612-13) anti-β-actin (1:10000; R1207-1) were obtained from Hangzhou HuaAn Biotechnology. The HRP-conjugated goat anti-mouse IgG secondary antibody (1:1000; CST, #91,196) and HRP-conjugated goat anti-rabbit IgG secondary antibody (1:1000; CST, #7074) were provided by Cell Signaling.

### RNA extraction and qRT-PCR

To isolate RNA from either CAFs or tissues, we employed the Trizol Universal Reagent (TIANGEN, DP424) and followed the manufacturer’s instructions. The reverse transcription reaction was then performed using the PrimeScript™ RT reagent Kit (Takara, RR037Q). The subsequent measurement of relative gene expression levels was carried out using the Premix Ex Taq™ (Probe qRT-PCR) kit (Takara, RR39LR). To ensure reliability and reproducibility, all experiments were conducted independently in triplicate. The qRT-PCR primers are provided in Supplementary Table S1.

### Cell migration assay

Cells at logarithmic growth phase were digested and re-suspended in serum-free medium. Cells were seeded at a concentration of 5 × 10^5^ cells/ml in the upper chamber of transwell plates containing 200 µL of medium. The lower chamber had 700 µL of 10% FBS-containing DMEM medium. The chambers were incubated at 37 °C for 24 h. Following the incubation, the chambers were immersed in 4% paraformaldehyde solution for 30 min, thereafter staining with crystal violet or DAPI. Cell counts on the undersurface of the PET membrane were determined by averaging cell counts obtained from the middle and four peripheral fields of perspective.

### EdU assay

The BeyoClick™ EdU-594 Cell Proliferation Assay Kit (Beyotime, C0078S) was used for detecting cell proliferation in this experiment. CAFs or NAFs cells (5 × 10^6^) were cultured overnight in a 6-well plate. On the following day, 2×EdU working solution was added to the cells in equal volume, bringing the final concentration to 10µM, and they were cultured for an additional 2 h at 37 °C. After EdU labeling, the medium was aspirated, and cells were fixed with 1 ml of 4% paraformaldehyde (Beyotime, P0099) at room temperature (RT) for 15 min. Next, the fixed cells were washed three times with 1 ml of washing buffer for 3–5 min each time, after which 1 ml of permeabilization solution (Beyotime, P0097) was added to each well and incubated at RT for 10–15 min. Then, the cells were washed 1–2 times with 1 ml of washing buffer, for 3–5 min each time, after removing the permeabilization solution. Next, 0.5 ml of Click reaction solution was added to each well, and the plate was gently shaken to ensure uniform reaction mixture coverage. Cells were incubated in the dark at RT for 30 min. Thereafter, the Click reaction solution was removed, and each well was washed three times with washing buffer, for 3–5 min each time. Lastly, the cells were examined and imaged using a fluorescence microscope with an excitation wavelength of 594 nm.

### Generation of stable expression mammalian cell lines by lentivirus

We generated a firefly luciferase plasmid and the KCNE4 gene for stable expression of the latter. Full-length human KCNE4 was amplified from cDNA of CAFs with the following primers: F: 5’-ATGGGACTGAAAATGGAGCCTCTGAA-3’, R: 5’-GGAATTCTGATGGATGTTCTCCGAGG-3’. For lentivirus packaging, three plasmid-packing systems were used. HEK293T cells were transfected with 800 ng of lentiviral vector, 400 ng of pCMV-VSV-G, and 600 ng of psPAX2 packaging plasmids, along with 1 mg/ml PEI (6 µl), in 6-well plates. After approximately 48–72 h, the cell medium containing lentiviruses was collected following filtration through a 0.22 μm filter membrane. Next, we infected CAFs and HCT116 cells with the KCNE4 lentivirus (MOI = 10) and firefly luciferase lentivirus (MOI = 10) for 48 h. The cells were subsequently selected with 1 µg/ml puromycin in medium for 7 days to obtain a stable pool of transgene-expressing cells. The harvested cells were then used for downstream experiments.

### RNA interference

The siRNAs were transfected into cells using GP-transfect-Mate (GenePharma, Shanghai, China) in serum-free medium following the manufacturer’ s instructions. Sequences of siRNAs used were as follows:

Si-NC: 5ʹ-UUCUCCGAACGUGUCACGUTT-3ʹ;

Si-KCNE4#1: 5ʹ-CCUCUUGGACUGGACGAUUTT-3ʹ;

Si-KCNE4#2: 5ʹ-CCUCCUGCUGCUGUACAAATT-3ʹ;

### Mouse models of peritoneal metastasis

Following a comprehensive database analysis, it was evident that KCNE4 exhibited no discernible correlation with gender or age. Employing methodologies akin to those described in previous literature [39], female BALB/C nude mice, weighing 15 g and aged 4–6 weeks, were deliberately housed under specific pathogen-free (SPF) conditions. This meticulous care and ethical approach adhered strictly to the guidelines set forth by the Institutional Animal Care and Use Committee at the Beijing Cancer Hospital (Ethics Approval Number: EAEC 2022-04). One million HCT116 cells were mixed with either 100,000 NAFs-OE Vector or 100,000 NAFs-OE KCNE4 and injected intraperitoneally to induce peritoneal metastasis. Luciferin suspended in PBS was injected intraperitoneally at a dose of 4 mg/mouse to visualize tumors using an In Vivo Imaging System. Approximately 4–6 weeks after transplantation, the tumor formation in the peritoneal metastasis nodes were quantified at the endpoint after sacrificing the mice.

### Mouse models of liver metastasis

Female BALB/C nude mice weighing 15 g and aged 4–6 weeks were housed in SPF conditions in compliance with the Institutional Animal Care and Use Committee guidelines of the Beijing Cancer Hospital (Ethics Approval Number: EAEC 2022-04). HCT116 cells and CAFs were cultured to approximately 80–90% confluence in a 10-cm dish and subsequently harvested with trypsin-EDTA solution (Catalog number GNM25200, Genom). The cells were washed twice with sterile PBS and enumerated. One million HCT116 cells were mixed with either 100,000 Vector-CAFs or 100,000 KCNE4-CAFs and injected into the spleen (50 µL per mouse) to induce liver metastasis. Tumors were visualized by injecting luciferin (intraperitoneal, 4 mg/mouse) suspended in PBS and imaged with an In Vivo Imaging System twice a week, approximately 8 weeks post-transplantation. To evaluate tumor formation in the spleen and nodal metastasis in the liver, mice were sacrificed at the endpoint, approximately 8 weeks post-transplantation.

### Immunohistochemistry (IHC)

The tissue blocks containing formalin-fixed and paraffin-embedded surgical specimens of esophageal cancer were sectioned into 4 mm slices for immunohistochemistry (IHC). Subsequently, the sectioned tissues underwent deparaffinization and were treated with 0.3% H_2_O_2_ in methanol at RT for 10 min to quench endogenous peroxidase activity. Antigen retrieval was accomplished by microwave heating in a sodium citrate buffer solution. Following cooling, sections were subjected to a 40-minute incubation with blocking reagent at RT. The sectioned tissues were then exposed to primary antibodies against anti-KCNE4 (1:100; 18289-1-AP) or anti-α-SMA (1:300; ET1612-13) overnight at 4℃. Subsequent to three 5-minute PBS washes, sections were incubated with secondary antibodies for 30 min at RT. Following further washing steps, visualization was achieved using an enzyme substrate, with subsequent counterstaining conducted with hematoxylin.

#### Hematoxylin and Eosin (H&E) staining

The FFPE (Formalin-Fixed Paraffin-Embedded) sections of mouse liver tissues underwent antigen retrieval via heat treatment at 65 °C for 1 h. Deparaffinization was achieved using xylene, followed by rehydration through graded ethanol solutions. Hematoxylin staining was applied for 5 min, and differentiation was performed using 1% hydrochloric acid in alcohol, followed by extensive tap water rinsing until achieving optimal blue coloration. Subsequently, counterstaining was executed with eosin. The sections were then dehydrated using ethanol, cleared with xylene for transparency, mounted with a neutral resin, and subjected to microscopic examination for imaging purposes.

### Statistical analysis

The data presented in this study represents the mean ± SEM (Standard Error of the Mean) and was analyzed using GraphPad Prism version 8 and R software (version 4.2.2; https://www.r-project.org/). Paired or unpaired Student’s t tests, one-way ANOVA tests, and Spearman correlation analysis were used to analyze bar graphs and assess correlations between two variables. Additionally, the log-rank test was applied to compare the Kaplan-Meier curves. The threshold for statistical significance was set at ****P < 0.0001, ***P < 0.001, **P < 0.01, *P < 0.05, or ns (*P* > 0.05).

## Results

### Co-expression network constructed by WGCNA

By utilizing multiple computational methods, including EPIC, xCell, MCP-counter, and TIDE, we successfully predicted CAF infiltration. Additionally, stromal scores were quantified using the estimate algorithm. Significantly, Kaplan-Meier analysis revealed that high levels of CAF infiltration and stromal scores are associated with poorer OS in CRC patients. The levels of CAF_EPIC, CAF_TIDE, and stromal scores were found to be markedly linked to worse OS in TCGA-CRC and GSE17536 datasets, underscoring the potential necessity of more thorough investigation of CAF and stroma-associated genes in CRC (Fig. S1a-f).

**Fig. 1:**
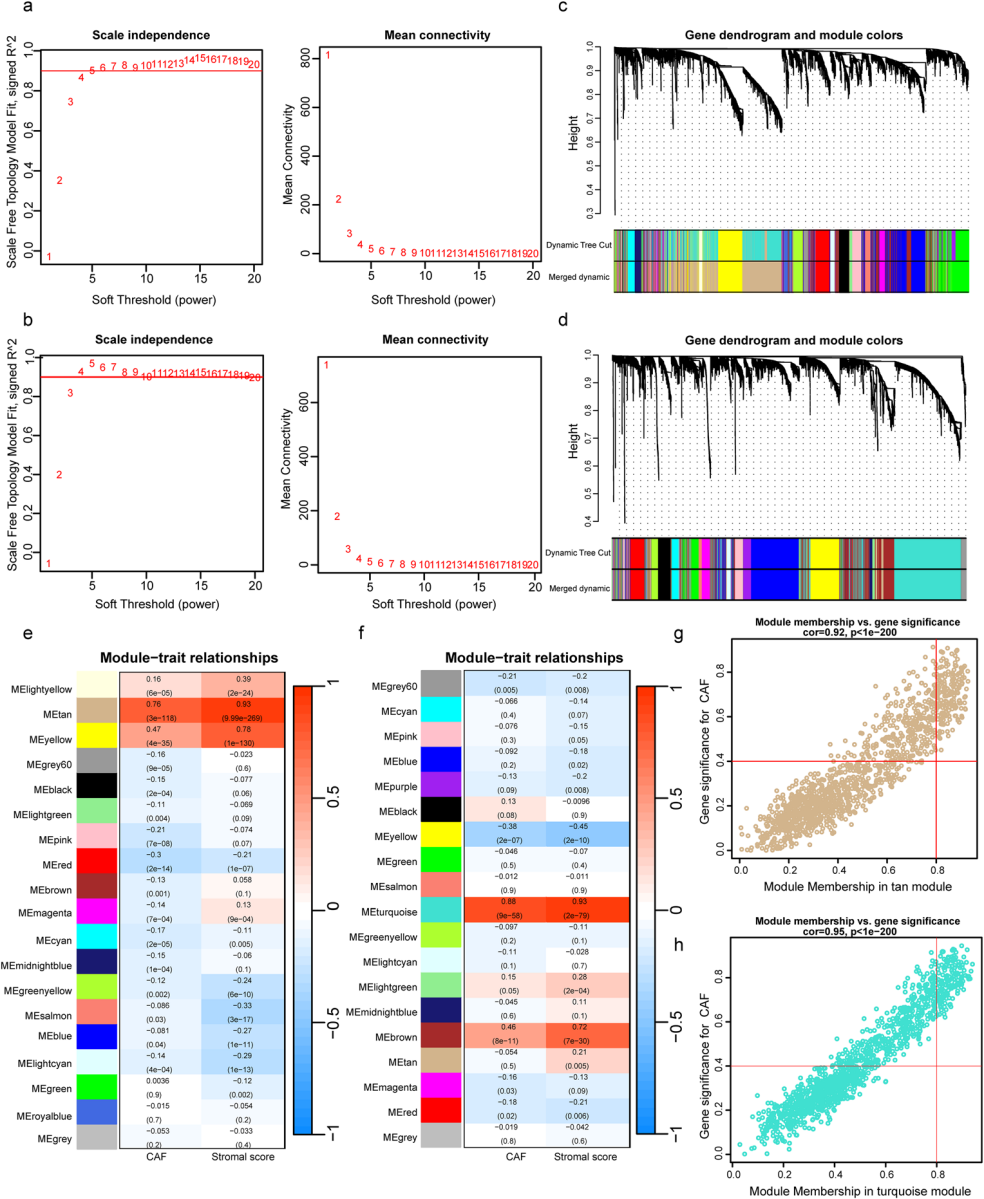
Co-expression network constructed by WGCNA. a, b. A soft-thresholding power (β) of 4 was chosen for subsequent analysis based on the scale-free topology criterion in TCGA-CRC (**a**) and GSE17536 (**b**). c, d. Genes with similar expression patterns were clustered into co-expression modules in TCGA-CRC (**c**) and GSE17536 (**d**) based on dendrograms created from hierarchical clustering. e, f. Correlation between gene modules’ eigengenes and phenotypes in TCGA-CRC (**e**) and GSE17536 (**f**) datasets. g, h. Scatter plots of the module membership (MM) and gene significance (GS) of each gene in the tan module of TCGA-CRC (**g**) and the turquoise module of GSE17536 (**h**)

WGCNA analysis was executed on both TCGA-CRC and GSE17536 datasets. To build a scale-free topology network, we determined the soft threshold power (β) of 4 in TCGA-CRC (scale-free R^2^ = 0.867) (Fig. 1a) and 4 in GSE17536 (scale-free R^2^ = 0.9292) (Fig. 1b). In TCGA-CRC, the hierarchical clustering tree identified 19 co-expression models (Fig. 1c), and the MEtan module displayed the strongest positive relationship the CAF proportion (Cor = 0.76, *P* = 3e-118) and stromal score (Cor = 0.93, *P* = 9.99e-269) (Fig. 1e). For GSE17536, the hierarchical clustering tree revealed that 19 co-expression models were clustered (Fig. 1d), with the MEturquoise module presenting the most significant positive correlation with the CAF proportion (Cor = 0.88, *P* = 9e-58) and stromal score (Cor = 0.93, *P* = 2e-79) (Fig. 1f). Thus, we directed our attention to these two modules for more research. In the MEtan module, the scatter plots suggested a robust relationship between MM and GS concerning CAF (Cor = 0.92, *p* = 1e-200) (Fig. 1g); in the MEturquoise module strong correlations were observed between MM and GS for CAF (Cor = 0.95, *P* = 1e-200) (Fig. 1h). Subsequently, applying MM > 0.8 and GS > 0.4 as the cut-off points, a total of 192 genes in the MEtan module of TCGA-CRC and 153 genes in the MEturquoise module of GSE17536 singled out as hub genes that were connected with CAF scores.

#### Functional analyses of selected CAF-related genes and construction a risk model based on CAF

**Fig. 2:**
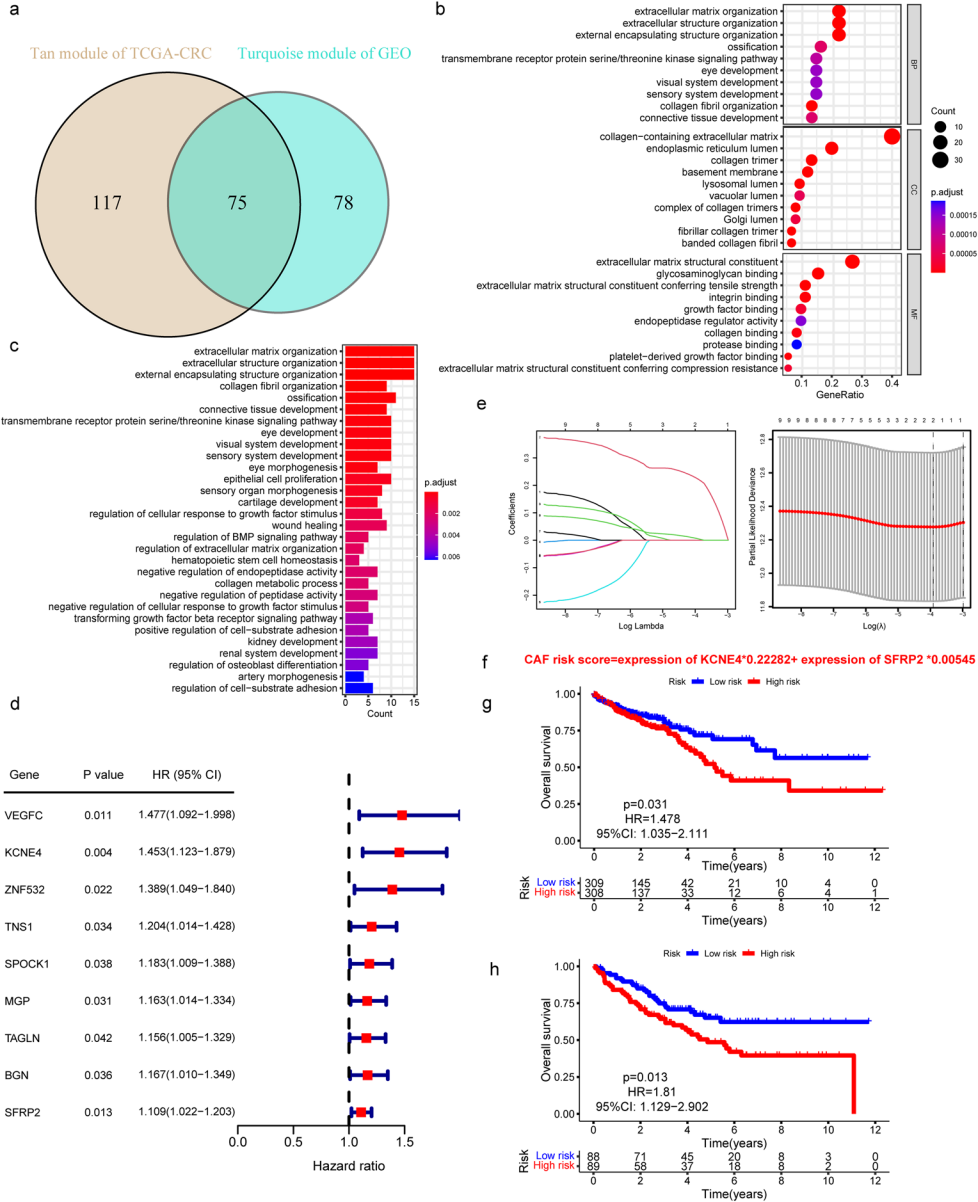
Identification of Key Genes and Establishment of Prognostic Models. (**a**) The intersection of TCGA-CRC tan and GSE17536 turquoise module genes was presented in the Venn diagram. (**b**, **c**). GO, KEGG analysis of the 75 genes. (**d**) Genes associated with overall survival in TCGA-CRC were screened using the univariate Cox analysis. (**e**) The two-gene prognostic signature was identified by Lasso–Cox regression analysis. (**f**) The construction of the CAF risk model. g, H. Kaplan-Meier analyses revealed that CRC patients in the high-CAF-risk group had poorer overall survival rates in both TCGA-CRC (**g**) and GSE17536 (**h**) cohorts

A Venn diagram was utilized to identify 75 hub genes by overlapping and screening the above-mentioned CAF-related genes (Fig. 2a). Thereupon, we investigated the functions and pathways associated with the 75 genes using GO and KEGG analyses. The main enriched GO terms were linked to extracellular matrix organization, extracellular structure organization (BP); collagen-containing extracellular matrix and extracellular matrix structural constituents were the major enriched CC and MF terms (Fig. 2b). The main enriched KEGG pathways were extracellular matrix organization, would healing, and regulation of BMP signaling pathway (Fig. 2c).

The 75 shared hub genes were analyzed using the univariate Cox regression, and 9 genes associated with OS with a p value less than 0.05 were identified and further analyzed using LASSO Cox regression (Fig. 2d, e). Ultimately, two genes were selected to construct the CAF risk model: CAF risk score = expression of KCNE4 * 0.22282 + expression of SFRP2 * 0.00545 (Fig. 2f). The patients stratified as high-CAF-risk using Kaplan-Meier curves showed poorer OS in comparison to those categorized as low-CAF-risk in both the TCGA-CRC cohort (HR = 1.478, 95% CI: 1.035–2.111, log-rank *p* = 0.031) (Fig. 2g) and GSE17536 cohort (HR = 1.81, 95% CI: 1.129–2.902, log-rank *p* = 0.013) (Fig. 2h).

### Correlations between CAF risk score and CAF infiltration, somatic variation, chemotherapy sensitivity and immunotherapy response

**Fig. 3:**
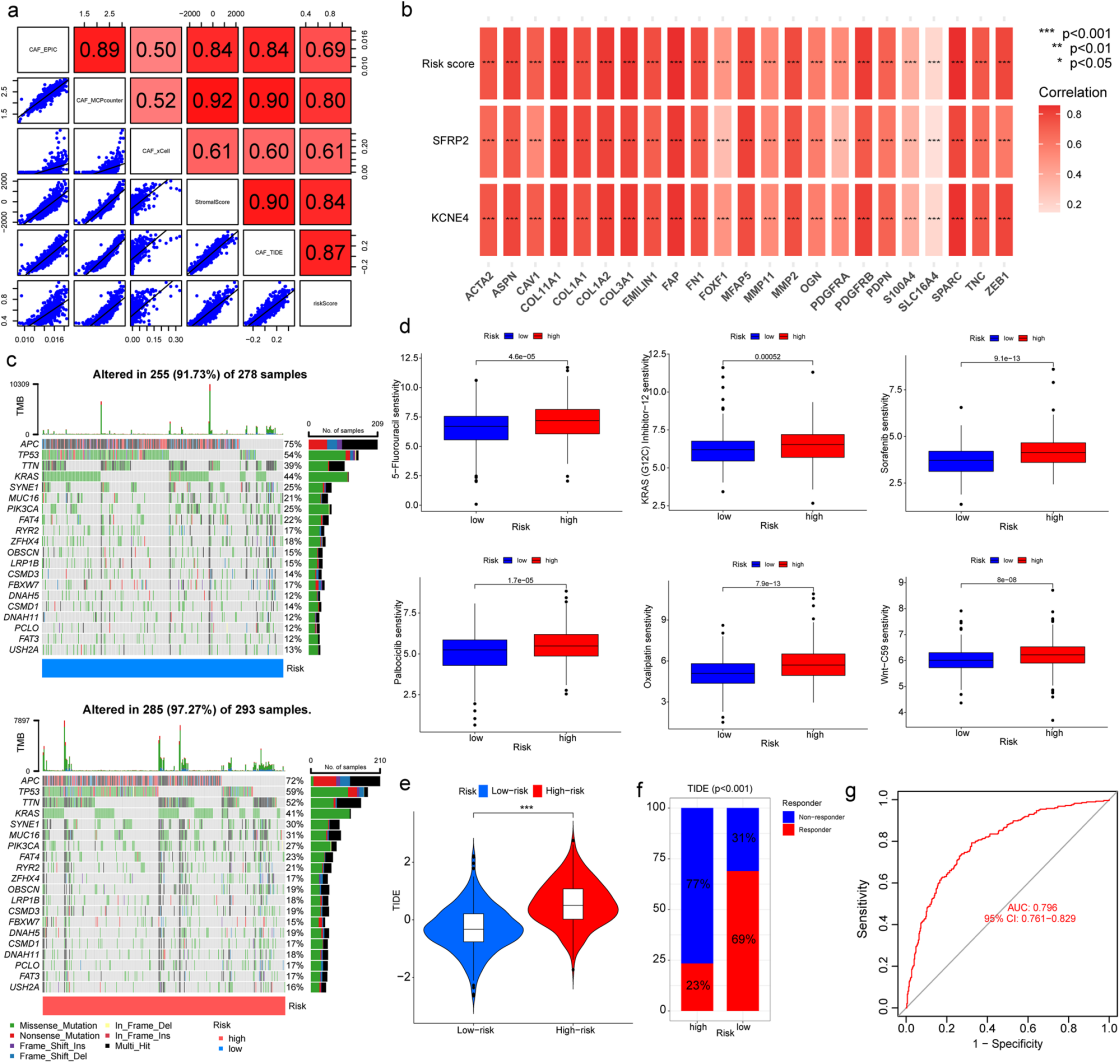
Correlations Between CAF risk score and Infiltration, somatic variation, and drug response. (**a**) Spearman’s correlation analysis of CAF risk score with stromal scores and multi-estimated CAF infiltration. (**b**) Spearman’s correlation analysis of CAF markers with CAF risk score and two signature genes. (**c**) The top 20 mutational genes in low- and high-CAF-risk groups of TCGA-CRC. (**d**) Comparison of chemotherapy drug IC50 values between low- and high-CAF-risk Groups. (**e**, **f**). TIDE immunotherapy prediction analyses. (**g**) ROC curves of the CAF risk score in predicting immunotherapy responses in TCGA-CRC

To verify the accuracy of the CAF model as a reliable predictor of CAF infiltrations, we performed Spearman’s correlation analyses between the CAF risk score and the stromal score, as well as the CAF abundances predicted by EPIC, xCell, MCP-counter, and TIDE, respectively. Our findings revealed a strong and positive correlation between the CAF risk score and the multi-estimated CAF infiltrations and stromal score in both TCGA-CRC (Fig. 3a). These results indicate that the CAF risk score is a credible predictor of CAF infiltrations. To further validate the association between the expression levels of the two genes and CAFs, we compared their expression levels with a collection of CAF markers in the TCGA-CRC cohort (Fig. 3b). A robust and positive association was detected between the expression levels of both genes and the majority of the CAF markers, indicating that the two genes are representative of CAFs.

We used waterfall plots to display the 20 genes with the highest mutational frequencies in the low- and high-risk subgroups, stratified by CAF risk score. Intriguingly, there was a considerable overlap in the most common mutational genes between the low- and high-CAF-risk groups (Fig. 3c). The finding indicated that there is not a significant correlation between the levels of CAF scores and the types of gene mutations present in CRC.

Typically, radical surgery is the primary modality of treatment for CRC patients, followed by adjuvant chemotherapy and ICI therapy where necessary. To assess the potential impact of CAF on CRC treatment, we estimated the half-maximal inhibitory concentration (IC50) values for multiple anti-tumor drugs, including those commonly used in CRC treatment, using the GDSC database. Wilcoxon analyses revealed significant differences in IC50 values observed between high- and low-CAF-risk patients with CRC. The results revealed that low-CAF-risk patients were sensitive to 5-Fluorouracil, KRAS (G12C) inhibitor-12, Sorafenib, Palbociclib, Oxaliplatin, and Wnt-C59 (Fig. 3d). Immunotherapy is considered one of the most significant recent advances, especially for patients with microsatellite instability-high (MSI-H) CRC. To assess the potential of the CAF risk score as a predictor of immunotherapy response in CRC patients, we employed the TIDE algorithm. Our analysis of TCGA-CRC dataset revealed that patients with low CAF scores showed greater immunotherapy sensitivity, as evidenced by lower TIDE scores, compared to those with high CAF scores (Fig. 3e). Additionally, the non-responder subgroup had significantly higher CAF scores than the responder subgroup (Fig. 3f). Our analysis of the TCGA-CRC dataset demonstrated that the model is an effective predictor of immunotherapy response, with an AUC value of 0.796 (Fig. 3g).

### Cross-dataset validation of important gene-KCNE4

**Fig. 4:**
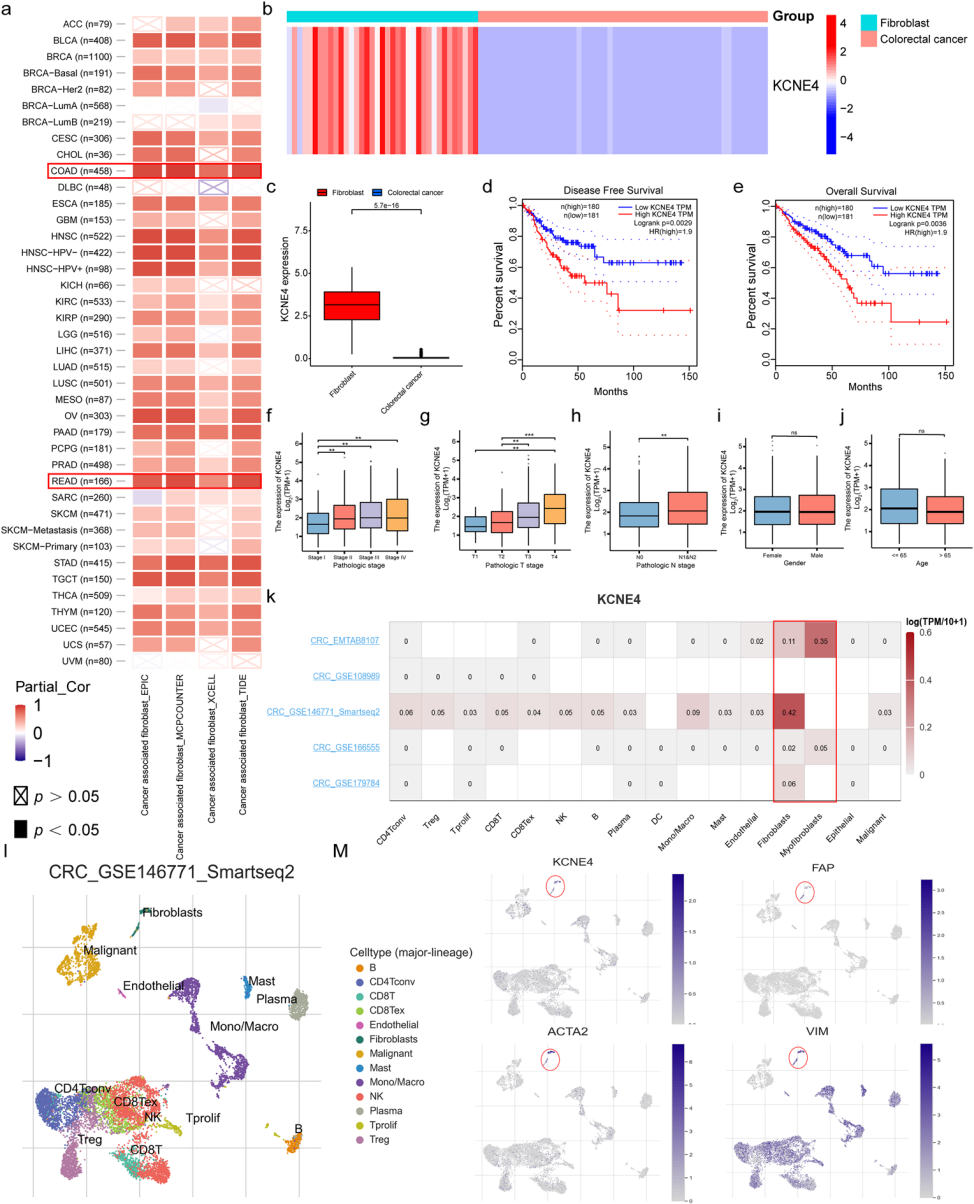
Cross-dataset validation of important gene-KCNE4. (**a**) The results of TIMER2.0 analysis reveal significant correlations between KCNE4 expression and the infiltration of CAFs in various tumor tissues, which are represented by red and blue squares on the scatterplot, where red denotes significant positive correlations, while blue denotes significant negative correlations. b, c. The expression levels of KCNE4 in CCLE database were analyzed using a heat map (**b**) and box plot (**c**). (**d**, **e**). The association between KCNE4 expression and OS as well as DFS in TCGA-CRC cohort. (**f**-**h**). The analysis also showed a positive correlation between KCNE4 expression levels and tumor stage. (**i**) The expression differences of KCNE4 in age status. (**j**) KCNE4 expression differences in gender. (**k**) The KCNE4 expression levels in different cell types using five single-cell sequencing datasets from the GEO database in CRC. (**l**, **m**). The relationship between KCNE4 expression levels and the expression of CAFs marker genes FAP, ACTA2, and VIM utilizing the single-cell dataset from CRC_GSE146771. Data in bar graphs indicate mean ± SEM. ns: no significant, **P < 0.01 and ***P < 0.001

The CAF risk model is predominantly based on two genes, KCNE4 and SFRP2. While SFRP2 has been extensively studied, the role of KCNE4 in fibroblasts remains unclear and has received limited attention in the literature. Therefore, the primary objective of our study is to elucidate the expression and distribution of KCNE4 within the microenvironment of CRC, as well as its role in fibroblasts. To investigate the association between KCNE4 expression and CAF infiltration in various cancer microenvironments, we utilized a range of algorithms, including EPIC, MCP-counter, XCell, and TIDE, available on the TIMER2.0 database, and obtained evidence demonstrating a positive correlation with a particular emphasis on CRC **(**Fig. 4a**)**. Subsequent analyses showed that the expression of KCNE4 in fibroblasts was significantly higher than that of CRC tissues **(**Fig. 4b, c**)**. We investigated the connection between KCNE4 expression and CRC prognosis by utilizing the GEPIA website; our observations indicated a negative correlation between KCNE4 expression levels and the OS and disease-free survival (DFS) of CRC patients **(**Fig. 4d, e**)**. Our investigation into the expression of KCNE4 in CRC revealed a positive correlation with tumor stage, and T and N classification, suggesting a strong link between elevated KCNE4 levels and poor clinical prognosis (Fig. 4f-h). In our subsequent analysis, we systematically examined the variations of this gene among male and female patients, as well as those below and above 65 years of age. Remarkably, no significant differences were discerned in the expression of KCNE4 between these two groups, underscoring the insignificance of this gene with respect to both age and gender (Fig. 4i, j). Moreover, we first analyzed the expression of KCNE4 at the single-cell levels in different cell types using the TISCH (Tumor Immune Single-cell Hub) database, which revealed that KCNE4 was exclusively highly expressed in fibroblasts and was low or not expressed in immune cells, epithelial cells, and tumor cells (Fig. 4k). Intriguingly, KCNE4 exhibited a co-expression pattern with characteristic CAF markers, including FAP, ACTA2, and VIM, within the CAFs group (Fig. 4l, m). Collectively, our findings suggest that KCNE4 is a specific marker of CAFs and is a negative prognosticator for CRC patients.

### KCNE4 tissue expression validation and CAF extraction in CRC

**Fig. 5:**
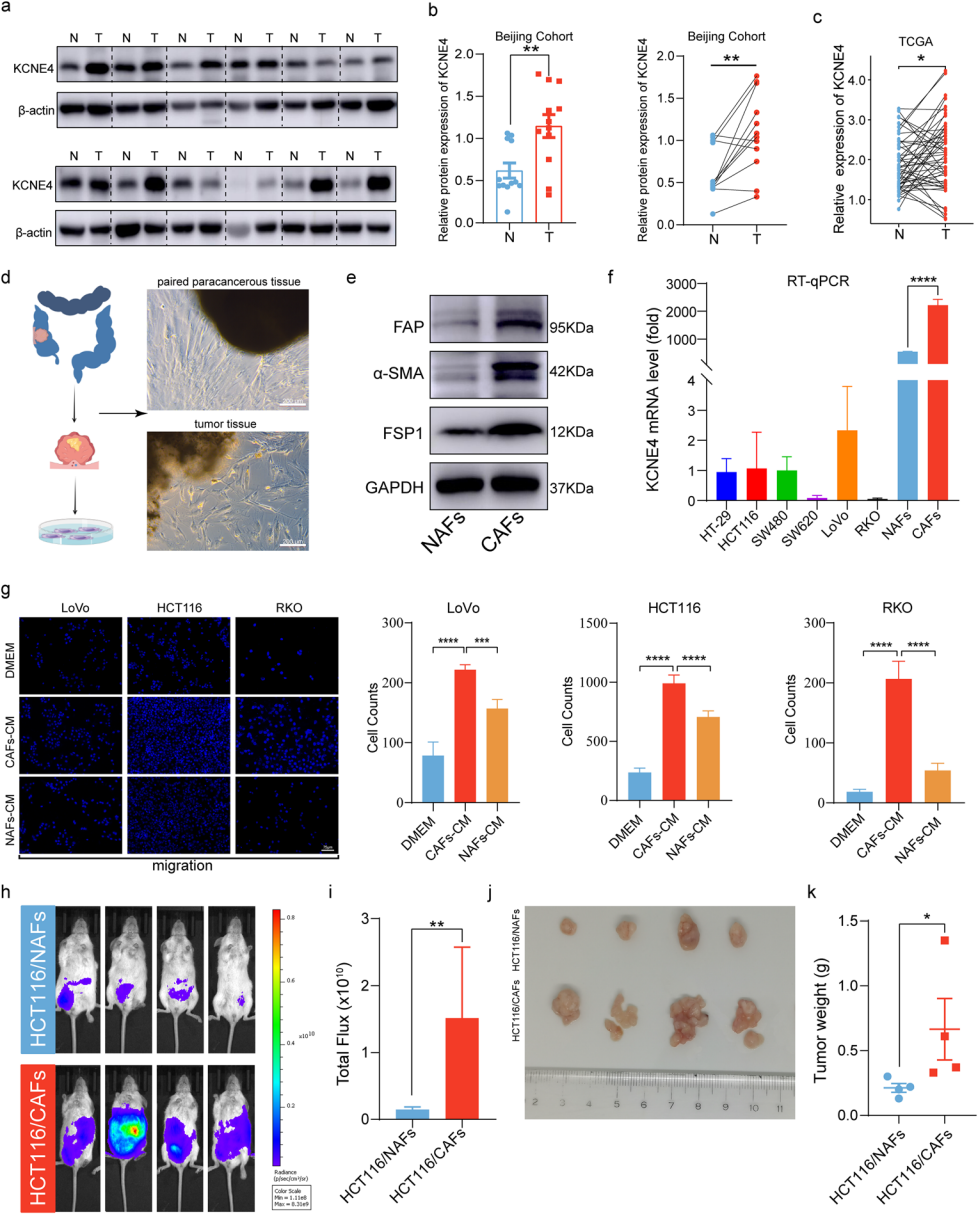
KCNE4 tissue expression validation and CAFs extraction in CRC. (**a**, **b**). KCNE4 protein expression was shown in 12 paired tumor and para-tumor tissue by Western blot. (“N” for non-tumor, “T” for tumor). (**c**) KCNE4 expression in paired CRC samples from TCGA. (**d**) Images display the isolation of NAFs and CAFs from CRC adjacent normal tissue and tumor tissue. (**e**) Western blot was used to measure the expression levels of FAP, α-SMA and FSP1 in NAFs and CAFs. (**f**) qRT-PCR analysis of KCNE4 in different cell types. (**g**) Migration of CRC cells incubated with CM derived from NAFs or CAFs. (**h**-**i**). In vivo bioluminescence imaging of mice administered with HCT116 (Luci) cells via intravenous intraperitoneal injection in the presence of either NAFs or CAFs (*n* = 4). (**j**) Dissected intraperitoneal tumor nodules after humanitarian execution (*n* = 4). (**k**) The intraperitoneal tumor weights in each group were statistically analyzed. Data in bar graphs indicate mean ± SEM. *P < 0.05, **P < 0.01, ***P < 0.001, ****P < 0.0001. Student’s t test (b, i, k), paired Student’s t test (c), multi-group analysis of variance (g)

We extracted proteins from both tumor and adjacent normal tissues from 12 CRC patients and quantified the expression level of KCNE4 using Western blotting. Our results indicated a significant upregulation of KCNE4 in tumor tissue compared to adjacent normal tissue (Fig. 5a, b), which were in line with TCGA-CRC paired analysis (Fig. 5c). To further elucidate the role of KCNE4 in CAF and NAF cells, we isolated and cultured these cell types from both tumor and adjacent normal tissues of CRC patients **(**Fig. 5d). We confirmed the successful isolation of CAF and NAF cells through Western blotting of various characteristic markers, including FAP, α-SMA, and FSP1. Notably, we observed significantly higher expression levels of these markers in CAF cells compared to NAF cells (Fig. 5e). The expression of KCNE4 was further analyzed in CRC cells, as well as NAFs and CAFs. Notably, we found that KCNE4 expression was primarily detected in fibroblasts and significantly higher than that in tumor cells. Remarkably, the expression of KCNE4 was higher in CAFs than in NAFs (Fig. 5f). Subsequently, to further confirm the tumor-promoting ability of CAFs, we applied conditioned media derived from NAFs and CAFs, along with regular media, to the bottom chamber of a transwell assay and observed their effect on tumor cell migration. Notably, compared to conditioned media from NAFs or regular media controls, media derived from CAFs significantly increased the migratory capacity of tumor cells (Fig. 5g). In order to further investigate the role of CAFs playing in promoting tumor progression, we performed in vivo experiments by co-culturing HCT116 cells with NAFs or CAFs, and then injecting the mixed cultures into the peritoneal cavity of mice to simulate peritoneal metastasis. Remarkably, our results showed that tumor spread was significantly increased in mice co-cultured with CAFs compared to those co-cultured with NAFs (Fig. 5h-k), which is consistent with our in vitro findings.

### The oncogenic role of KCNE4 in CAFs

**Fig. 6:**
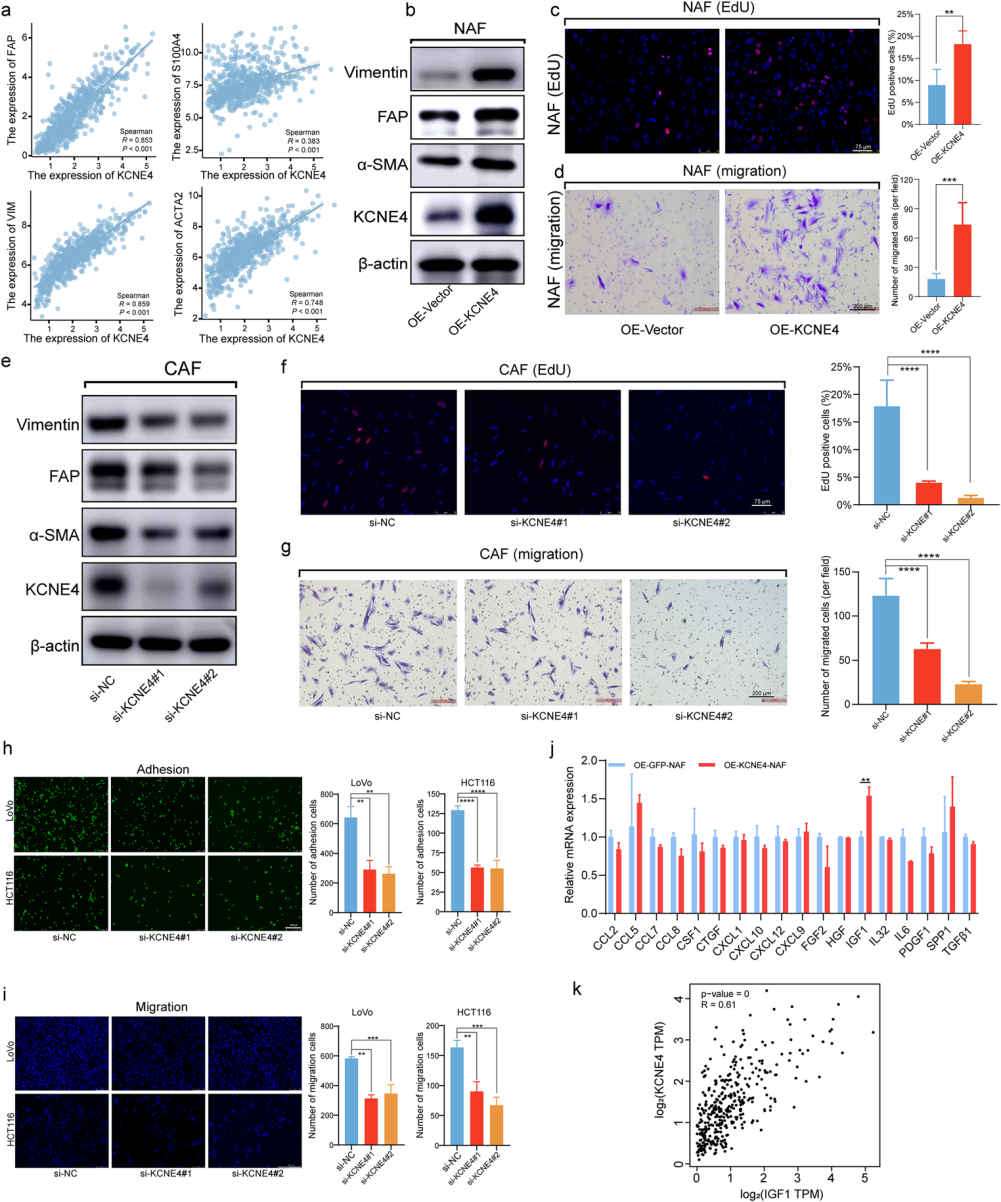
The oncogenic role of KCNE4 in CAFs. (**a**) Spearman’s correlation analysis was utilized to determine the correlation between the expression levels of FAP, VIM, FSP1, ACTA2, and KCNE4, with the data from the GEPIA database. (**b**) Immunoblot Analysis of CAFs Markers in NAFs with Induced KCNE4 overexpression. (**c**) The effect of KCNE4 on NAFs proliferation was determined by EdU incorporation assay. (**d**) Impact of KCNE4 on NAFs migration revealed via transwell Migration. (**e**) The protein levels of CAFs markers in CAFs transfected with KCNE4 siRNAs. (**f**) EdU assay for detecting proliferation CAFs Proliferation following KCNE4 siRNA transfection. (**g**) Migration of CAFs transfected with KCNE4 siRNAs. (**h**) Cell adhesion assay of LoVo- and HCT116-GFP cells on CAFs transfected with KCNE4 siRNA-NC or siRNA-KCNE4. (**i**) Migration of LoVo and HCT116 cells incubated with CM derived from CAFs transfected with KCNE4 siRNA-NC or siRNA-KCNE4. (**j**) The mRNA expression levels of the target genes of multiple chemokine and cytokine. (**k**) Analysis of the correlation between KCNE4 and IGF1 from the TCGA database. Data in bar graphs indicate mean ± SEM. **P < 0.01, ***P < 0.001, ****P < 0.0001. Student’s t test (c, d), multi-group analysis of variance (f, g, h, i, j)

To investigate the functional role of KCNE4 expression in CAFs, we conducted a correlation analysis between KCNE4 and several malignancy markers in CAFs, including FAP, FSP1, VIM, and ACTA2, using the TCGA database. Notably, our results demonstrated a strong positive correlation between KCNE4 and these malignancy markers, indicating an important role for KCNE4 in the malignant phenotype of CAFs (Fig. 6a). According to previous studies, CAF migratory capability, attributed to specific migratory, adhesive, and paracrine signaling mechanisms, may be a significant contributor to cancer progression and metastasis [40, 41]. Moreover, we conducted EdU incorporation and migration assays and found that KCNE4 overexpression significantly increased NAFs proliferation and migration (Fig. 6b-d). In contrast, when KCNE4 was knocked down in CAFs, we observed a downregulation of CAFs malignancy markers, as well as a significant reduction in CAFs proliferation and migration, as demonstrated by EdU incorporation and migration assay results (Fig. 6e-g).

GSEA was performed on the TCGA-CRC dataset between high- and low-CAF-risk groups. As displayed in Fig. S2a, b, the major enriched KEGG signaling pathways were cell adhesion molecules cams, ECM receptor interaction and focal adhesion. Extensively, ssGSEA results declared that the CAF risk score was positively correlated with focal adhesion and TGF-β signaling hallmarker gene sets (Fig. S2c-f). Considering these results, we established a in vitro co-culture model. CAFs with overexpression or knockdown of KCNE4 gene were seeded in culture plates until full confluence. Afterwards, GFP-labeled HCT116 or LoVo cells were added onto the CAFs monolayers. After 15 min, unbound tumor cells were washed away and the remaining adherent tumor cells were analyzed. CAFs in the control group exhibited stronger adhesion to tumor cells compared to those with KCNE4 knockdown (Fig. 6h). Meanwhile, overexpression of KCNE4 in CAFs enhanced adhesion between CAFs and tumor cells (Fig. S3a). In addition, we added conditioned media from fibroblasts with knockdown or overexpression of KCNE4 in the lower chamber of transwell plates. Our results demonstrated that conditioned media from KCNE4-knockdown CAFs weakened the migration-promoting effects of CAFs on tumor cells (Fig. 6i). In contrast, conditioned media from KCNE4-overexpressing NAFs enhanced the migration-promoting effects on tumor cells (Fig. S3b). In our pursuit of understanding the pivotal functions mediated by KCNE4 and its interaction with chemotactic and cytokine factors, we delved into the existing literature on CAFs secretome. Conducting a thorough correlation analysis between genes reported in these studies and our KCNE4, we identified a subset of genes exhibiting strong correlations. These selected genes underwent further validation through qRT-PCR testing. Notably, our qRT-PCR results unveiled a significant and pronounced upregulation of IGF1 in the NAFs-OE KCNE4 group compared to the NAFs-OE Vector group (Fig. 6j). Furthermore, through in-depth analysis of TCGA database, we elucidated a discernible positive correlation between IGF1 and KCNE4, thus substantiating our experimental observations (Fig. 6k). These findings suggested that KCNE4 played a crucial role in enhancing adhesion between fibroblasts and tumor cells, as well as facilitating tumor cell migration by promoting fibroblast self-activation.

### Upregulation of KCNE4 in CAFs drives metastasis of CRC cells

**Fig. 7:**
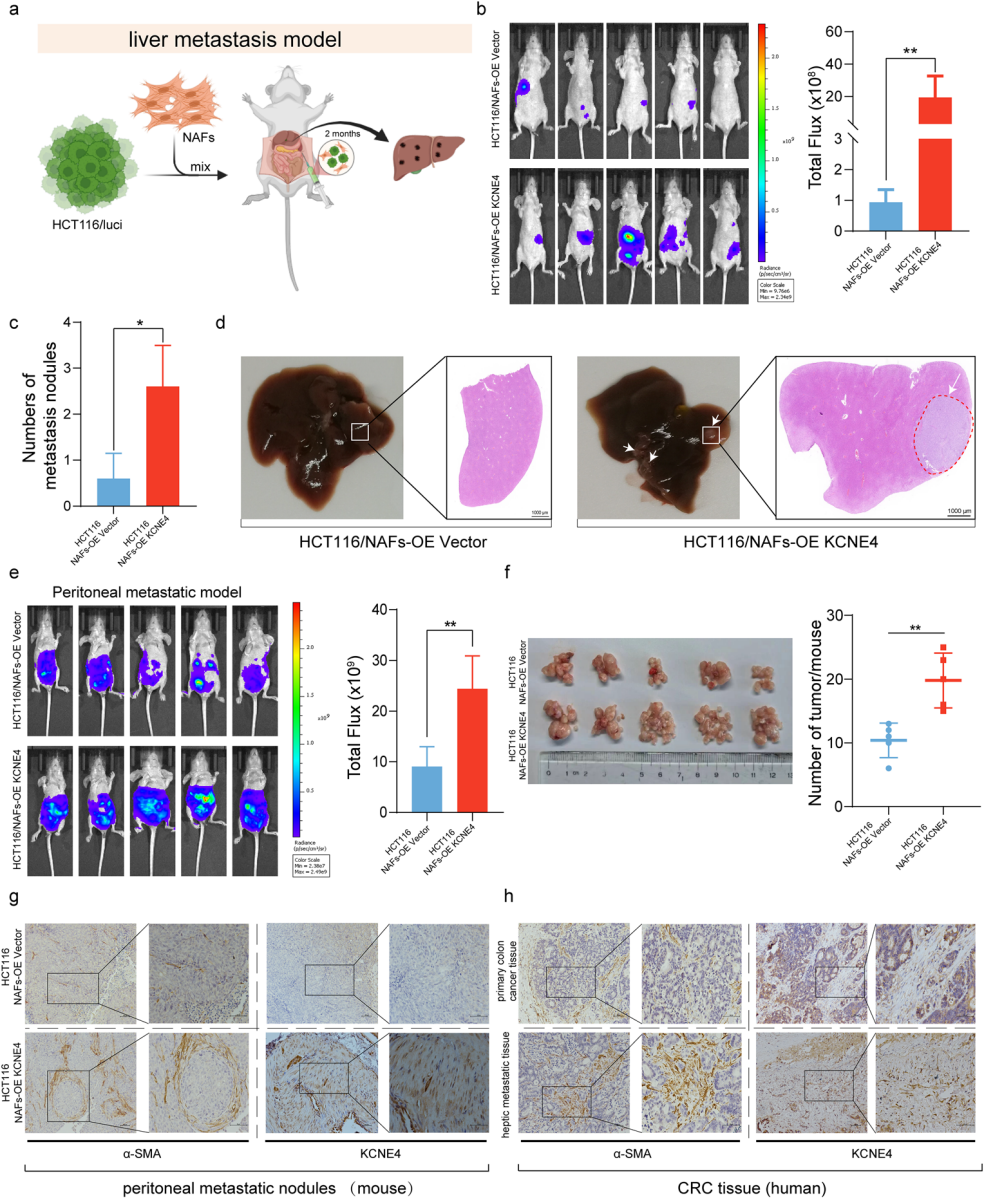
Upregulation of KCNE4 in CAFs drives liver metastasis of CRC cells. (**a**) A mouse liver metastasis model was constructed by simultaneous injection of CRC cells and NAFs into the spleen. (**b**) In vivo bioluminescence imaging of mice administered with HCT116 (Luci) cells via intrasplenic injection in the presence of either NAFs-OE Vector or NAFs-OE KCNE4 (*n* = 5). (**c**) Comparison of liver metastases in HCT116/NAFs-OE Vector and HCT116/NAFs-OE KCNE4 groups. (**d**) Representative HE staining reveals liver metastasis in nude mice following 8 weeks of spleen injection with CRC cells and NAFs-OE Vector or NAFs-OE KCNE4 (*n* = 5). The white arrow indicates the metastatic node (scale bar, 1000 μm). (**e**) In vivo bioluminescence imaging of mice administered with HCT116 (Luci) cells via intraperitoneal injection in the presence of either NAFs-OE Vector or NAFs-OE KCNE4 (*n* = 5). (**f**) Comparison of intraperitoneal metastases in HCT116/NAFs-OE Vector and HCT116/NAFs-OE KCNE4 groups. (**g**) Immunohistochemistry was employed to assess the distribution of KCNE4 and α-SMA in peritoneal metastatic nodules derived from mice in Fig. 7f. (**h**) Immunohistochemistry was employed to scrutinize the distribution of KCNE4 and α-SMA in paired primary colorectal cancer tumors and their corresponding liver metastases. Data in bar graphs indicate mean ± SEM. *P < 0.05, **P < 0.01. Student’s t test (b, c, e, f)

The most common metastasis seen in CRC patients is liver metastasis. Hence, in order to determine the impact of elevated KCNE4 expression in NAFs on liver metastasis of CRC cells, we conducted rigorous in vivo assays by injecting NAFs and CRC tumor cells into the spleens of nude mice (Fig. 7a). Remarkably, our findings unveiled a compelling phenomenon, as the overexpression of KCNE4 in NAFs remarkably facilitated the metastatic potential of CRC cells (Fig. 7b). Notably, this remarkable enhancement in metastatic capability led to the formation of a significantly higher number of metastatic colonies within the murine liver (Fig. 7c). In each experimental group, liver slices were stained with hematoxylin-eosin (HE), revealing noticeable tumor metastatic foci in the liver in the NAFs overexpressing KCNE4 (Fig. 7d).

In addition, we implemented an intraperitoneal metastasis model in mice. HCT116 cells were introduced into the mouse peritoneal cavity, co-administered with either NAFs-OE Vector or NAFs-OE KCNE4 cells. Strikingly, mice in the NAFs-OE KCNE4 group exhibited a significantly higher number of intraperitoneal metastatic nodules compared to the NAFs-OE Vector group (Fig. 7e, f). Subsequently, we conducted histological staining on the peritoneal nodules, revealing a pronounced elevation in the expression of α-SMA and KCNE4 in the NAFs-OE KCNE4 group compared to the NAFs-OE Vector group (Fig. 7g).

Furthermore, our validation efforts extended to the clinical domain. Directly obtaining primary CRC lesions and their paired liver metastatic lesions from patients, we performed immunohistochemical staining for α-SMA and KCNE4. The results demonstrated a substantial increase in the expression of α-SMA and KCNE4 in the liver metastatic lesions compared to the primary lesions (Fig. 7h).

## Discussion

CRC is a heterogeneous disease with multiple subtypes that possess distinct molecular and clinical characteristics [42]. Although the molecular mechanisms driving the development and progression of CRC have been extensively studied, the specific role of CAFs in this subtype remains uncertain [43, 44]. CAFs are critical constituents of the TME and have been shown to promote CRC growth and progression [45]. Consistent with previous findings, our study revealed that patients with high stromal or CAFs scores had poorer prognosis [46, 47]. By applying WGCNA, univariate Cox and LASSO regression algorithms, a two-gene (SFRP2 and KCNE4) prognostic CAF model was constructed and validated [48]. In addition, by employing the TIDE online algorithm, we found a significant correlation between lower CAFs risk scores and improved response to immunotherapy in patients with CRC.

Further exploring the two identified key genes, we found limited research on the KCNE4 gene in the context of CAFs. We subsequently utilized CRC single-cell sequencing data from the GEO database to confirm that KCNE4 is predominantly expressed in CAFs within tumor tissues and its expression is coincident with that of malignant CAFs markers. GSEA revealed that ECM receptor interaction and focal adhesion gene sets were highly enriched in the high-CAF-risk group. CAFs and cancer cells could communicate with each other through direct contact during tumor progression, such as cell adhesion. Our previous study reported that CDH11-mediated juxtacrine interaction of gastric cancer cells and fibroblasts promotes metastasis via YAP/tenascin-C [49]. Mechanistically, we noted that knockdown of KCNE4 in CAFs resulted in a downregulation of malignant CAF markers, a decrease in adhesion between CAFs and tumor cells, and a reduction in CAF-mediated pro-metastatic effects on tumor cells. Conversely, overexpression of KCNE4 yielded opposite results. These results suggest that KCNE4 represents a promising therapeutic target for CAFs in cancer treatment.

With respect to the two identified markers in the model, SFRP2 was reported as a top biomarker of a predominant CAF population and inactivation of SFRP2 in CAFs impaired their ability to induce the migration and invasion of colon cancer cells, as well as their tumorigenicity in vivo by creating an immunosuppressed environment [48]. Mo et al. reported that SFRP2^+^ CAFs are crucial for predicting GC patients’ prognosis and the efficacy of immune checkpoint blockade therapy, as well as promoting GC progression [50]. We observed that high-CAF-risk CRC patients were less sensitive to several drugs like ICI therapy, and this result fitted well with the finding that the aforementioned study in GC.

KCNE4 was previously demonstrated to modulates Kv1.3 current properties, including slowing activation, accelerating inactivation, and retaining the channel at the endoplasmic reticulum [51]. Despite the importance of ion channel regulation in tumorigenesis [52], the role of KCNE4 in cancer remains poorly understood. Encouragingly, a recent study investigating CRC found that elevated KCNE4 expression was associated with radioresistance and upregulation of the PI3K/AKT signaling pathway [53]. Our study revealed that KCNE4 was predominantly expressed in CAFs and had a pro-tumorigenic function. Expression analysis of primary cultured CAFs and tumor cell lines revealed a significantly higher expression of KCNE4 in CAFs than in tumor cells. Further investigation is needed to determine the relative importance of KCNE4 in tumor cells versus CAFs.

The underlying mechanisms driving the transition from NAFs to CAFs phenotype remain inadequately understood. According to Chen et al. study [52], the TME is characterized by an elevated potassium-rich milieu, which has been shown to impair the anti-tumor potential of tumor-associated macrophages (TAMs). Given this, it is plausible that elevated KCNE4 expression in CAFs represents an adaptation to the potassium-rich milieu of the tumor immune microenvironment. In the future, the role of potassium ion dynamics in modulating the transition from NAFs to CAFs, as well as the specific mechanisms by which elevated KCNE4 expression regulates fibroblast malignant phenotypes, represent key areas of investigation that will deepen our understanding of the complex interplay between the TME and cancer cells. Elucidating these mechanisms may be critical for the development of targeted therapeutic strategies aimed at disrupting tumor-stromal interactions and improving clinical outcomes for CRC patients.

## Conclusions

In summary, we established a two-gene prognostic CAF signature which held great potential for predicting patient survival outcomes and response to therapeutic interventions. Meanwhile, our study provided compelling evidence indicating that CRC CAFs played a critical role in promoting tumor growth and metastasis, and that elevated expression of KCNE4 was associated with enhanced proliferation and migratory capacity of CAFs, as well as enhanced tumor-promoting properties of CAFs. These findings highlighted the importance of the TME in promoting cancer progression and suggested that targeted disruption of tumor-stromal interactions, such as those mediated by KCNE4, could represent a promising strategy for the development of novel anti-cancer therapeutics.

### Electronic supplementary material

Below is the link to the electronic supplementary material.

Supplementary Material 1 Fig. S1: Kaplan–Meier analyses of CRC patients. Kaplan-Meier plot of overall survival in TCGA-CRC (a–c) and GSE17536 (d–f) stratified by CAF infiltrations and stromal scores

Supplementary Material 2 Fig. S2: Gene set enrichment analysis of KEGG. a-b. Ten representative enriched KEGG pathways by GESA. c-f. ssGSEA results showed CAF risk score was positively correlated focal adhesion, ECM receptor interaction, TGF-β signaling pathway and regulation of actin cytoskeleton enrichment scores

Supplementary Material 3 Fig. S3: Enhanced tumor cell adhesion and metastasis-promoting effects of KCNE4 overexpressing CAF. a. Cell adhesion assay of LoVo- and HCT116-GFP cells on CAFs transfected with OE-Vector or OE-KCNE4 plasmid. b. Migration of LoVo and HCT116 cells incubated with CM derived from CAFs transfected with OE-Vector or OE-KCNE4 plasmid. Data in bar graphs indicate mean ± SEM. *P < 0.05, **P < 0.01, ***P < 0.001. Student’s t test (a, b)

Supplementary Material 4 Table S1: Primers for qRT-PCR

Supplementary Material 5 Raw data

## Data Availability

All data presented and analyzed during this study are included in this article.

## Abbreviations

ANOVA: Analysis of variance
BPs: Biological processes
CAFs: Cancer-associated fibroblasts
CCLE: Cancer Cell Line Encyclopedia
CCs: Cellular components
CRC: Colorectal cancer
DFS: Disease-free survival
EMT: Epithelial–mesenchymal transition
EPIC: Estimate the Proportion of Immune and Cancer
ESTIMATE: Employed the Estimation of Stromal and Immune cells in Malignant Tumor tissues
FBS: Fetal bovine serum
GDSC: Genomics of Drug Sensitivity in Cancer
GEO: Gene Expression Omnibus
GSEA: Gene Set Enrichment Analysis
HEK293T: Human Embryonic Kidney 293T
HR: Hazard ratio
iCAFs: Inflammatory CAFs
ICI: Immune checkpoint inhibitor
KCNE4: Potassium Voltage-Gated Channel Subfamily E Regulatory Subunit 4
LASSO: Least absolute shrinkage and selection operator
MCP-counter: Microenvironment cell populations-counter
MFs: Molecular functions
MSI-H: Microsatellite instability-high
myoCAF: smyofibroblast CAFs
NAFs: Normal associated fibroblasts
OS: Overall survival
qRT-PCR: quantitative Real-time PCR
SEM: Standard Error of the Mean
SPF: Specific pathogen-free
TAMs: Tumor-associated macrophages
TCGA: The Cancer Genome Atlas
TME: Tumor microenvironment
TOM: Topological overlap measure
WGCNA: Weighted gene co-expression network analysis
RT: Room temperature
HE: Hematoxylin-eosin
