# Supplementary figures and images for: Influential upregulation of KCNE4: Propelling cancer associated fibroblasts-driven colorectal cancer progression
Source: Cancer Cell Int. 2024 Mar 10;24:103. doi: 10.1186/s12935-024-03274-9 (PMC10926681; doi:10.1186/s12935-024-03274-9)

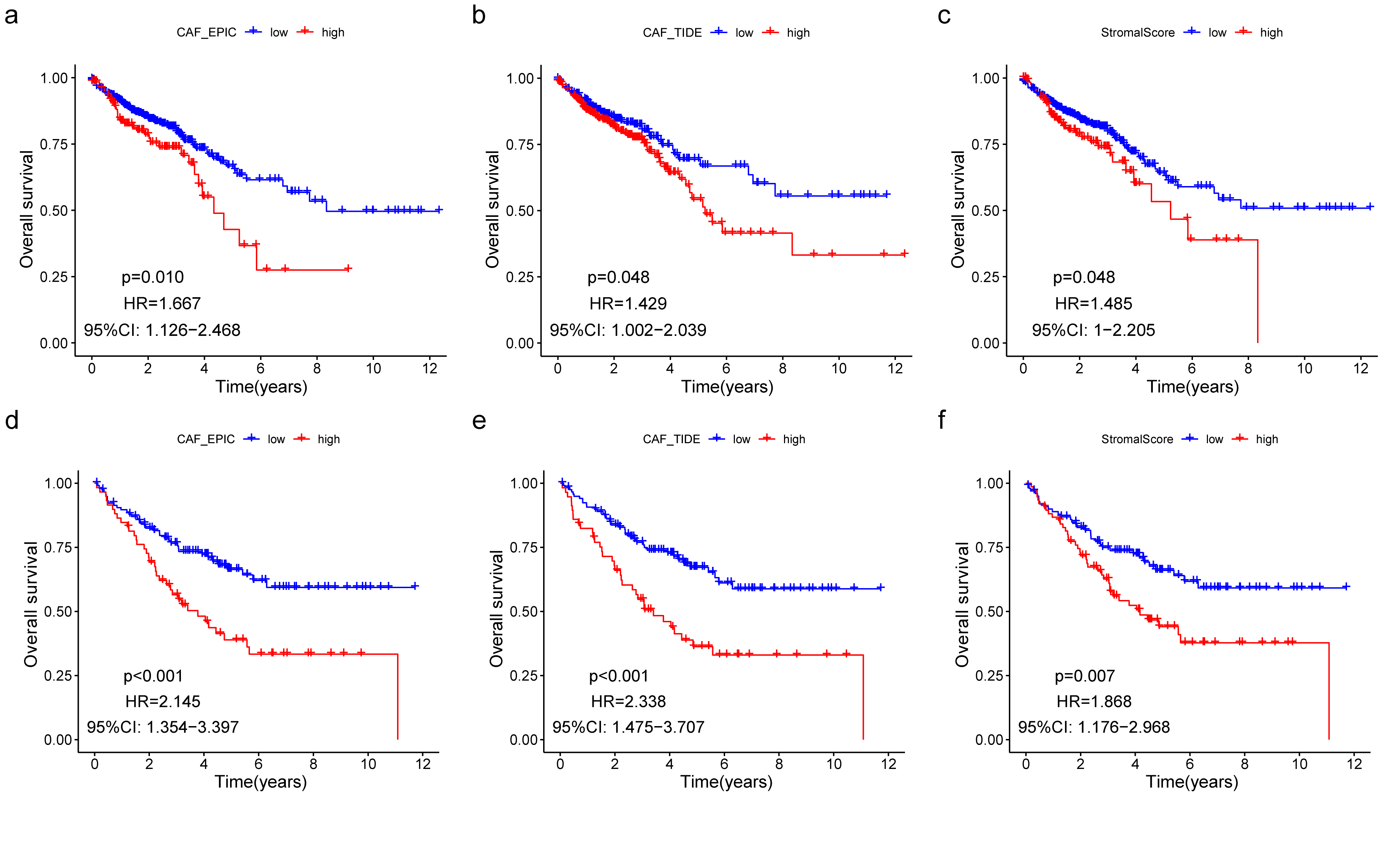

Supplement: Supplementary file 1 — Supplementary Material 1 Fig. S1: Kaplan–Meier analyses of CRC patients. Kaplan-Meier plot of overall survival in TCGA-CRC (a–c) and GSE17536 (d–f) stratified by CAF infiltrations and stromal scores [file 12935_2024_3274_MOESM1_ESM.tif]

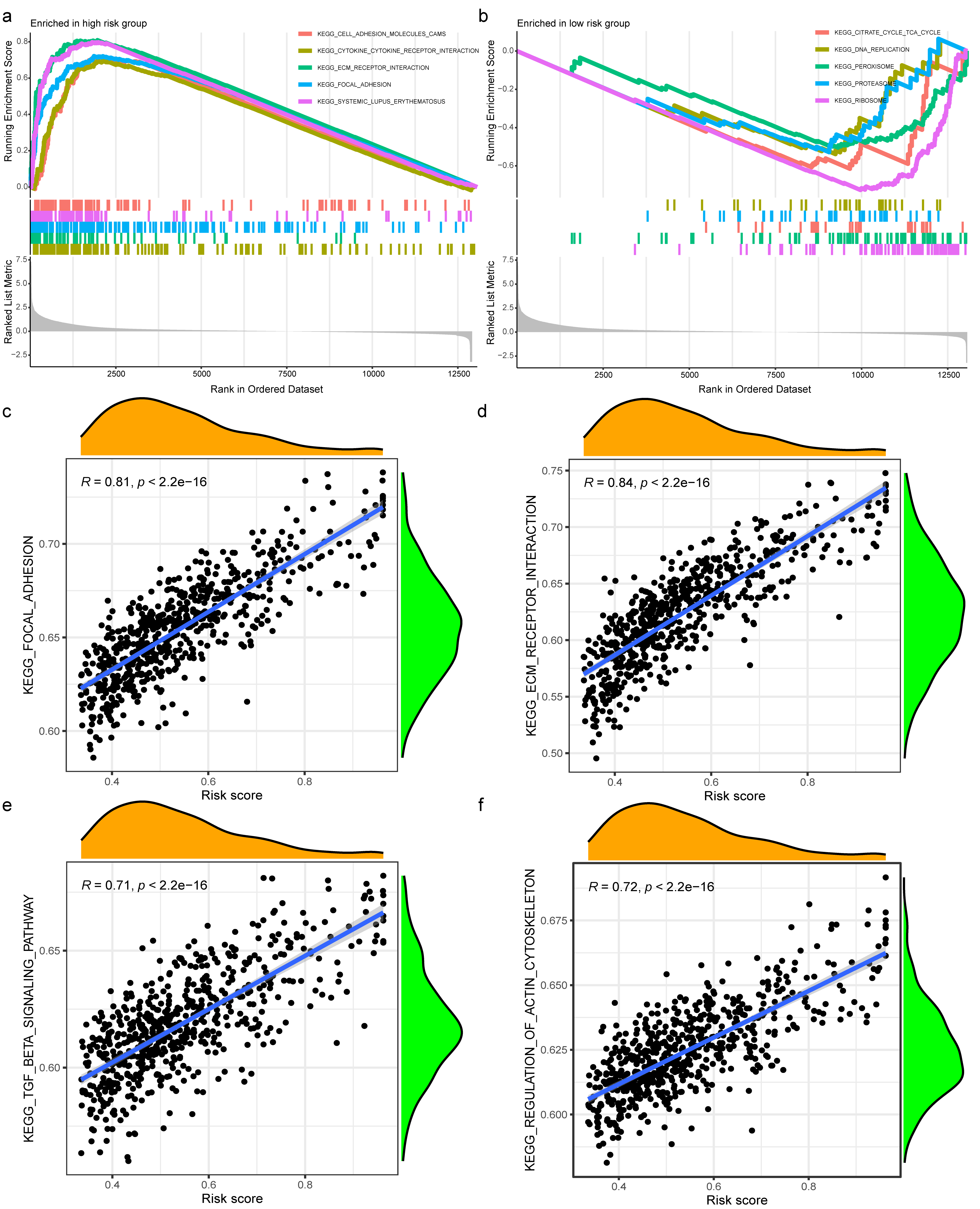

Supplement: Supplementary file 2 — Supplementary Material 2 Fig. S2: Gene set enrichment analysis of KEGG. a-b. Ten representative enriched KEGG pathways by GESA. c-f. ssGSEA results showed CAF risk score was positively correlated focal adhesion, ECM receptor interaction, TGF-β signaling pathway and regulation of actin cytoskeleton enrichment scores [file 12935_2024_3274_MOESM2_ESM.tif]

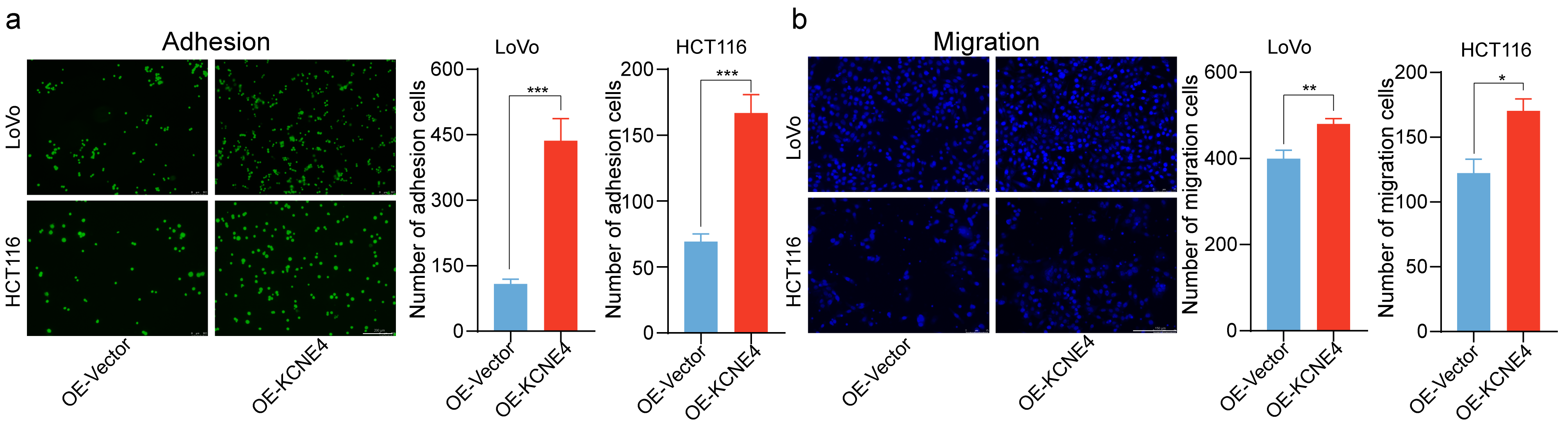

Supplement: Supplementary file 3 — Supplementary Material 3 Fig. S3: Enhanced tumor cell adhesion and metastasis-promoting effects of KCNE4 overexpressing CAF. a. Cell adhesion assay of LoVo- and HCT116-GFP cells on CAFs transfected with OE-Vector or OE-KCNE4 plasmid. b. Migration of LoVo and HCT116 cells incubated with CM derived from CAFs transfected with OE-Vector or OE-KCNE4 plasmid. Data in bar graphs indicate mean ± SEM. *P < 0.05, **P < 0.01, ***P < 0.001. Student’s t test (a, b) [file 12935_2024_3274_MOESM3_ESM.tif]
