# Supplementary material for: Influential upregulation of KCNE4: Propelling cancer associated fibroblasts-driven colorectal cancer progression
Source: Cancer Cell Int. 2024 Mar 10;24:103. doi: 10.1186/s12935-024-03274-9 (PMC10926681; doi:10.1186/s12935-024-03274-9)

Unedited blot and gel images

Full unedited blots for Figure 5a

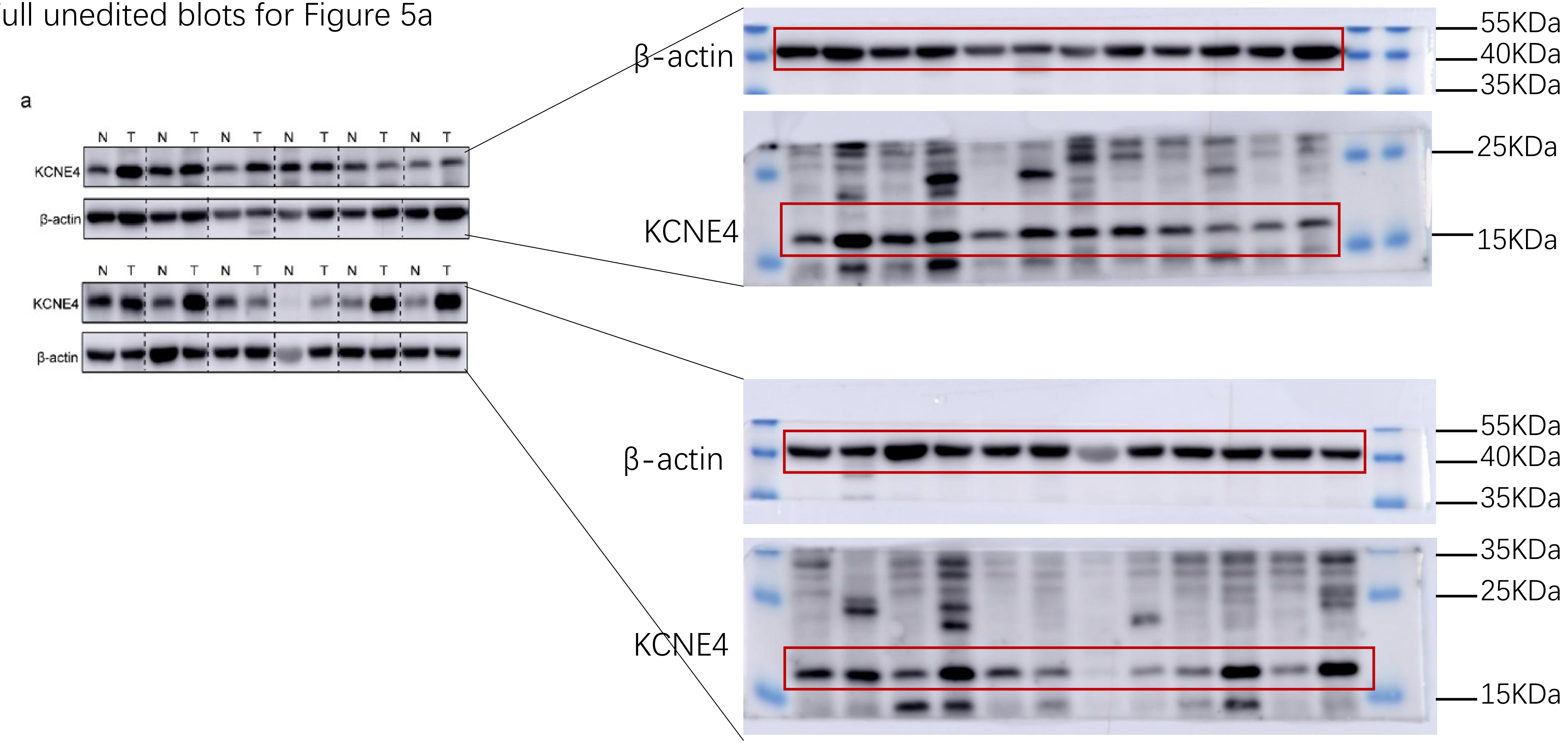

Unedited blot and gel images

Full unedited blots for Figure 5e

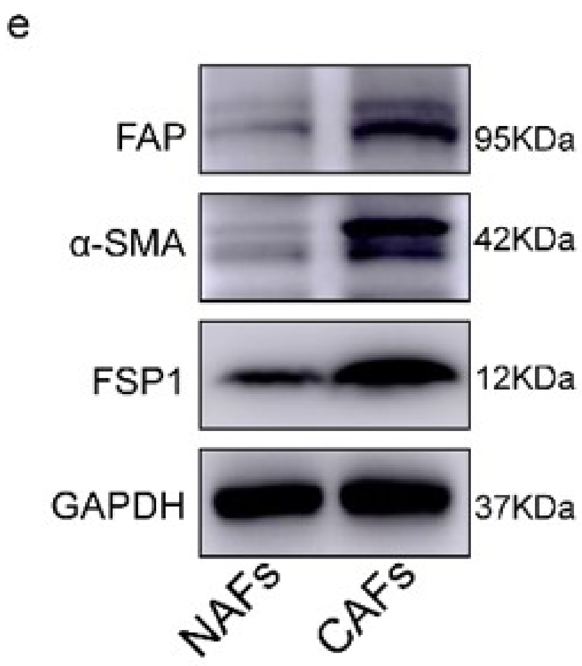

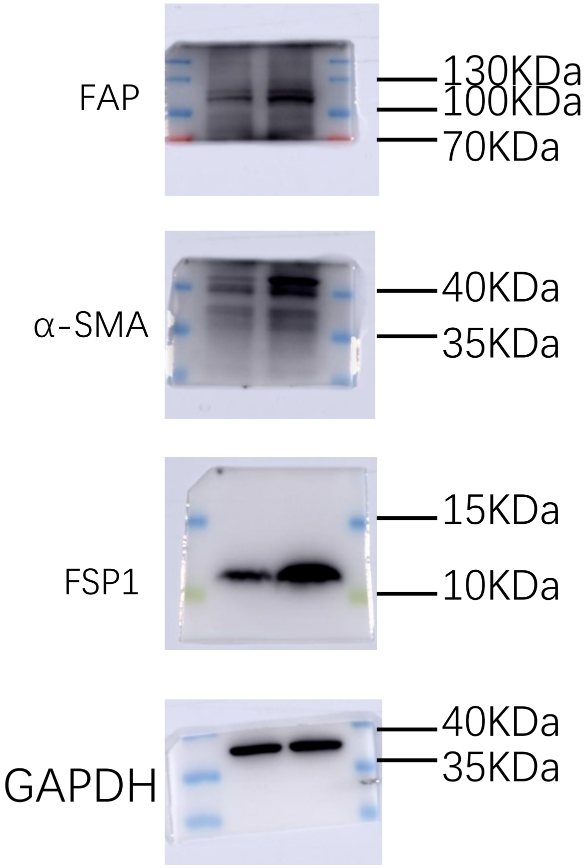

Unedited blot and gel images

Full unedited blots for Figure 6b

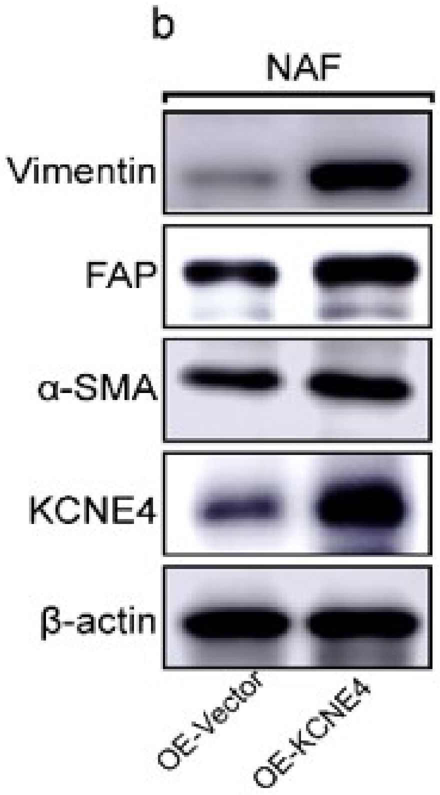

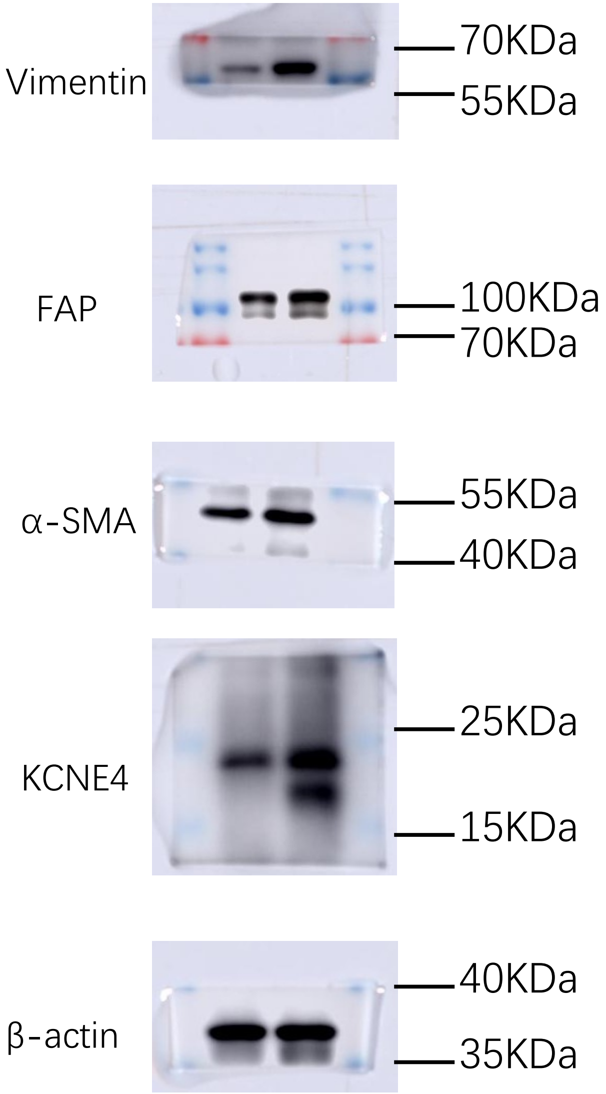

Unedited blot and gel images

Full unedited blots for Figure 6e

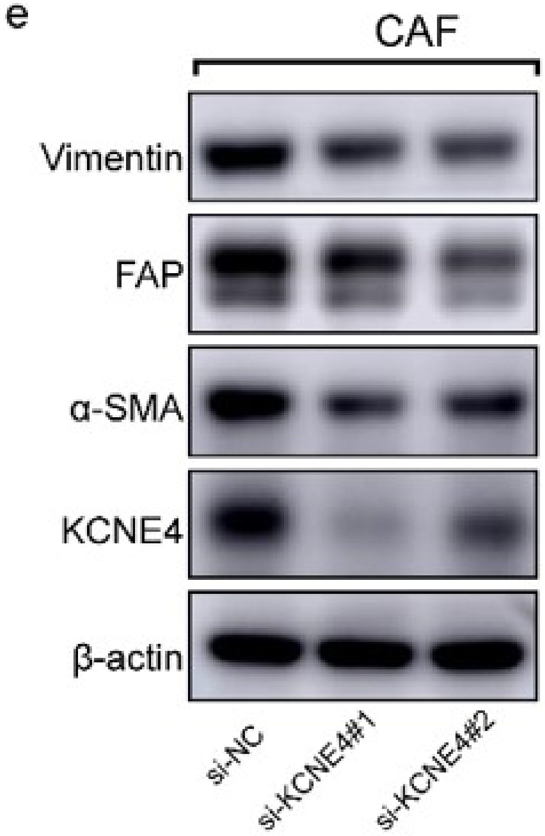

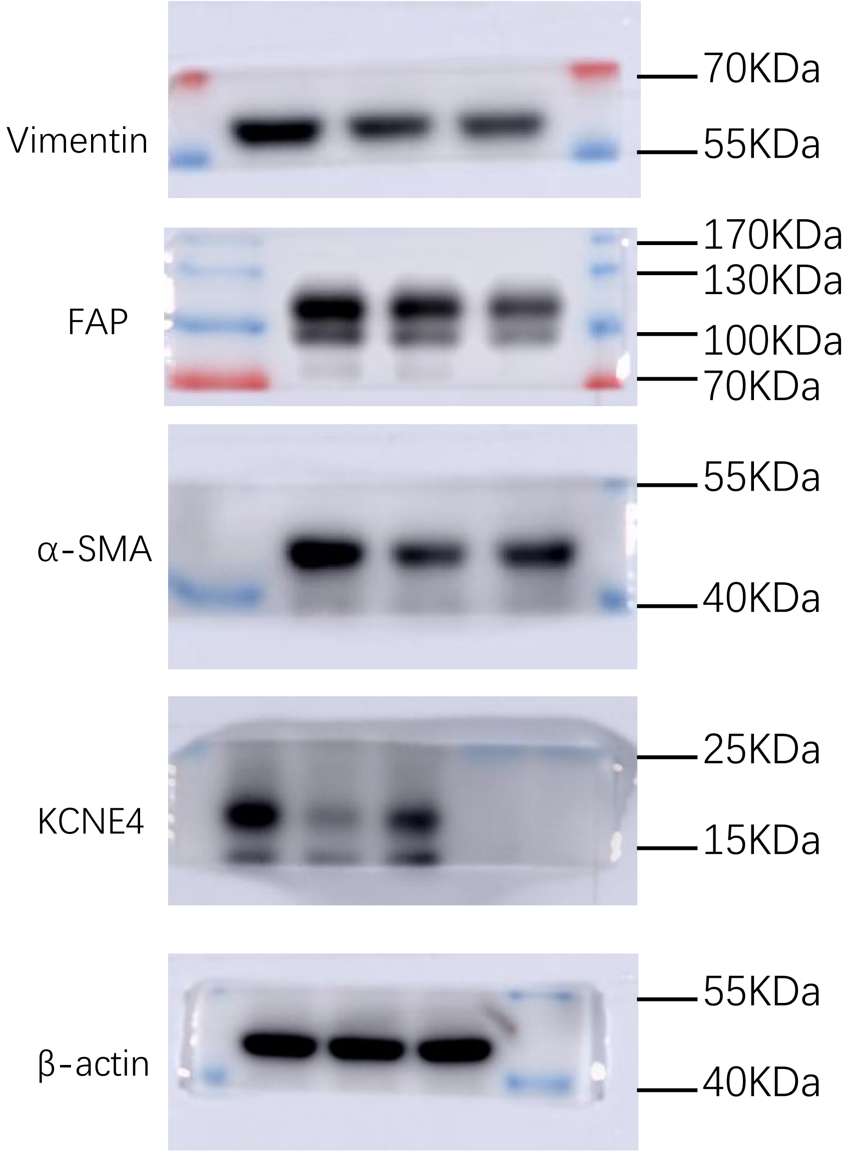

Supplement: Supplementary file 5 — Supplementary Material 5 Raw data [file 12935_2024_3274_MOESM5_ESM.pdf]
