# Supplementary material for: Influential upregulation of KCNE4: Propelling cancer associated fibroblasts-driven colorectal cancer progression
Source: Cancer Cell Int. 2024 Mar 10;24:103. doi: 10.1186/s12935-024-03274-9 (PMC10926681; doi:10.1186/s12935-024-03274-9)
Supplement: Supplementary file 4 [file 12935_2024_3274_MOESM4_ESM.odt]

Table. S1: primers for qRT-PCR

| Primers name | Sequence (5’-3’) |
| --- | --- |
| ACTIN-F | GAACGGTGAAGGTGACAGCA |
| ACTIN-R | GTAACAACGCATCTCATATTTGGAA |
| CCL2-F | CAGCCAGATGCAATCAATGCC |
| CCL2-R | TGGAATCCTGAACCCACTTCT |
| CCL5-F | TCATTGCTACTGCCCTCTGC |
| CCL5-R | CCAGACTTGCTGTCCCTCTC |
| CCL7-F | CACACAGAAGTGGGTCCAGG |
| CCL7-R | CACTCTGAGAAAGGACAGGGT |
| CCL8-F | ATGCTGAAGCTCACACCCTT |
| CCL8-R | GCAGCAGGTGATTGGAATGG |
| CSF1-F | AAGTTTGCCTGGGTCCTCTC |
| CSF1-R | AGACCAACAACAGCAGGGAG |
| CTGF-F | AAAAGTGCATCCGTACTCCCA |
| CTGF-R | CCGTCGGTACATACTCCACAG |
| CXCL10-F | GTGGCATTCAAGGAGTACCTC |
| CXCL10-R | TGATGGCCTTCGATTCTGGATT |
| CXCL12-F | ATTCTCAACACTCCAAACTGTGC |
| CXCL12-R | ACTTTAGCTTCGGGTCAATGC |
| CXCL1-R | GGTAGCCCTTGTTTCCCCC |
| CXCL1-R | GGTAGCCCTTGTTTCCCCC |
| CXCL9-F | CCAGTAGTGAGAAAGGGTCGC |
| FGF2-F | AGAAGAGCGACCCTCACATCA |
| FGF2-R | CGGTTAGCACACACTCCTTTG |
| HGF-F | GCTATCGGGGTAAAGACCTACA |
| HGF-R | CGTAGCGTACCTCTGGATTGC |
| IGF1-F | GCTCTTCAGTTCGTGTGTGGA |
| IGF1-R | GCCTCCTTAGATCACAGCTCC |
| IL32-F | TGGCGGCTTATTATGAGGAGC |
| IL32-R | CTCGGCACCGTAATCCATCTC |
| IL-6-F | GGTACATCCTCGACGGCATCT |
| IL-6-R | GTGCCTCTTTGCTGCTTTCAC |
| KCNE4-F | ATGGAGCCTCTGAACAGCAC |
| KCNE4-R | GTAGCCCAGCATGATTCCGA |
| PDGF1-F | GCAAGACCAGGACGGTCATTT |
| PDGF1-R | GGCACTTGACACTGCTCGT |
| SPP1-F | CACCTGTGCCATACCAGTTA |
| SPP1-R | TGTGTGCCTTTTTGTCCAAGC |
| TGFB1-F | CTAATGGTGGAAACCCACAACG |
| TGFB1-R | TATCGCCAGGAATTGTTGCTG |
